# Ethnobotany in a Modern City: The Persistence in the Use of Medicinal Plants in Guadalajara, Mexico

**DOI:** 10.3390/plants14172788

**Published:** 2025-09-05

**Authors:** Rosa Elena Martínez-González, Francisco Martín Huerta-Martínez, Cecilia Neri-Luna, Lucía Barrientos-Ramírez, Alejandro Muñoz-Urias

**Affiliations:** 1 Departamento de Botánica y Zoología, Doctorado en Ecofisiología y Recursos Genéticos, Centro Universitario de Ciencias Biológicas y Agropecuarias, Universidad de Guadalajara, Cam. Ramón Padilla Sánchez 2100, Las Agujas, Zapopan 44600, Jalisco, Mexico; rosa.mgonzalez@academicos.udg.mx; 2 Departamento de Ecología, Centro Universitario de Ciencias Biológicas y Agropecuarias, Universidad de Guadalajara, Cam. Ramón Padilla Sánchez 2100, Las Agujas, Zapopan 44600, Jalisco, Mexico; cecilia.neri@academicos.udg.mx (C.N.-L.); alejandro.munozu@academicos.udg.mx (A.M.-U.); 3 Instituto de Madera Celulosa y Papel, Centro Universitario de Ciencias Exactas e Ingenierías, Universidad de Guadalajara, Cam. Ramón Padilla Sánchez 2100, Las Agujas, Zapopan 44600, Jalisco, Mexico; lucia.barrientos@academicos.udg.mx

**Keywords:** secondary metabolites, medicinal plants, biological activity, principal coordinate analysis, ethnopharmacology

## Abstract

The traditional use of medicinal plants around the world has a long history, predominantly in low- and middle-income countries. Previous ethnobotanical research pertaining to urban environments demonstrated that the legacy of the use of medicinal plant species persists worldwide; however, information about the main city in the occidental part of Mexico is scarce regarding this traditional knowledge and its variation during the last few decades. A database was created from interviews with local people who had inhabited the oldest neighborhoods of Guadalajara for at least 30 years and by using different electronic databases. In addition, the correct taxonomic identification of species was supported via corroboration through local and other digital herbariums. Furthermore, a Principal Coordinate Analysis (PCoA) was performed on the database information to search for relationships among the medicinal plant species used. An inventory of 137 medicinal plants was created, where the plant species most commonly used in the five old neighborhoods of Guadalajara City were muicle (*Justicia spicigera* Schltdl.), pirul (*Schinus molle* L.), manzanilla (*Matricaria chamomilla* L.), valeriana (*Valeriana* sp.), calabaza (*Cucurbita pepo* L.), cola de caballo (*Equisetum arvense* L.), tepezcohuite (*Mimosa tenuiflora* Poir.), salvia (*Salvia officinalis* L.), canela (*Cinnamomum verum* J. Presl.), tila estrella (*Tilia americana* var. *mexicana* (Schltdl.) Hardin), cedrón (*Aloysia citrodora* Paláu), uva (*Vitis vinifera* L.), jengibre (*Zingiber officinale* Roscoe) and gobernadora (*Larrea tridentata* (DC.) Coville). Illnesses of the cardiovascular, digestive, urinary, respiratory, nervous, muscular and reproductive systems, as well as culture-bound syndromes, were mostly treated with these plant species. Moreover, *J. spicigera*, *M. chamomilla* and *L. tridentata* were used for eight medical purposes, followed by *Z. officinale* with five medicinal practices. In contrast, only two medicinal uses were recorded for *C. pepo*, *M. tenuiflora* and *S. officinale*. The PCoA explained 65.88% of the variation accumulated at the first three ordination axes and formed four groups of species, which were related to their geographical origin. Eight of the fourteen species that are commonly used as medicinal plants are from America, and the rest come from Europe and Asia. This study confirms the persistence of traditional knowledge related to medicinal plants, and the diseases empirically addressed among the inhabitants of Guadalajara City are common in other parts of the world and in different regions of Mexico. These findings are supported by electronic databases that comprise multiple studies related to the phytochemical compounds and medical validation regarding their biological activity, supporting the empirical use and efficacy of these medicinal plants.

## 1. Introduction

Although scientific knowledge about the number of plant species on the planet is still lacking, around 35,000 medicinal species are estimated. People have a long history with the traditional use of medicinal plants around the world [1]. In fact, in ancient civilizations across all continents, it is possible to find traces of their utilization. According to data from the World Health Organization (WHO), about 75% of people use herbal medicinal plant extracts for primary health care worldwide. Therefore, interest in natural products as a source of new medicines has increased at the industrial and research levels, where about 40% of modern drugs in use have been developed from natural products. Since these natural products have wide structural diversity—mainly comprising small molecules (<2000 Da) called secondary metabolites, which the body can quickly absorb and metabolize—their interactions have biological effects that benefit health [1]. About 39% of the drugs approved between 1983 and 1994 were natural products or their derivatives, including 60–80% antibacterial and anticancer agents from a natural source [2].

Therefore, despite the progress in pharmacology, plants are still used therapeutically, especially in developing countries [3]; moreover, this practice is highly actively promoted [1]. In recent decades, scientific interest has turned again to medicinal plants as a result of research on their traditions, compounds and compositions, which might reveal new opportunities to address future challenges and essential medicinal requirements—not only in the form of “soft therapeutics” but also as a source of effective resources for the treatment of illnesses, including serious diseases, with unsatisfactory therapeutic solutions [4].

Mexico ranks second worldwide in the traditional use of medicinal plants, where approximately 4500 plants are used for medicinal purposes [5]. Furthermore, medicinal plant species, either native or introduced, are preferred by over 90% of Mexicans due to their effectiveness, affordability and fewer side effects compared to allopathic medicines [6]. An invaluable example of this traditional knowledge dates back to 1552 and is included in the “Libellus de Medicinalibus Indorum herbis”, also known as the “De la Cruz-Badiano Codex” or “Little Book on Indigenous Medicinal Herbs”, which represents the first catalog of Nahuatl indigenous medicine; it also contains a mixture of pictographic and alphabetic elements from pre-Hispanic and colonial Mexico [7]. It seems likely that the floristic richness in this country (25,077 species of vascular plants) [8], together with its great cultural diversity (68 indigenous languages with 30 variants) [9], has led to the plants’ traditional use as medicine since ancient times. Mexico’s significance has encouraged numerous studies about medicinal plants in the area over several decades, including classical ethnobotanical studies [10,11,12,13] and papers about potential phytochemicals [6] as new alternatives to face the increase in disease prevalence (such as cancer, diabetes, and gastrointestinal problems) or as alternatives for drug-resistant illnesses and medicinal validations [14].

For centuries, the oral transmission process was considered essential to preserve the customs, knowledge and beliefs linked to herbal medicine, particularly in connection to indigenous communities. Thus, oral transmission among generations has been widely documented around the world. In Mexico, there are relevant ethnobotanic papers related to 67% of the 56 ethnic groups of Mexico, particularly from the south-central region [15,16], south areas like Oaxaca [17,18] and Chiapas [19], and northeastern Mexico [20].

Nevertheless, some concern exists about the loss of traditional knowledge related to plant species useful for human health, particularly in contemporary cities or big urban areas, since traditional oral transmission has been modified due to several factors (i.e., migration processes, gentrification, loss of intergenerational communication, and inclusion of natural products from other countries based on exotic plant species). In addition, habitat degradation due to the city’s expansion limited the availability of plants and resulted in local customs being left behind [21,22]. Therefore, in this context, urban ethnopharmacology is rapidly advancing and gaining significant attention throughout the world, particularly for the increasing number of people that will reside in urban environments in coming years [23]. Also, each city has its own dynamic environment that favors the circulation and consumption of knowledge and plants through formal and informal circuits [24]. Several urban areas around the world, including in North and South America, Europe, Africa, Asia and Australia, have growing scrutiny toward the use of medicinal plants; however, the legacy of medicinal plant use persists. Furthermore, its value is increasing as it expands the availability of health care and provides other essential products, particularly for underprivileged populations [25].

However, despite the advances in this field, the topic remains marginal, and increased attention must be paid to the “ethnobiospheres in urban environments” [23]. Urban botanical knowledge is a complex system that varies according to the pluricultural context and internal biocultural variation in the diversity of cities [24]. Following this line of exploration in Mexico, there is published information about city markets in Puebla [26], Queretaro [27] and Hidalgo [28]. Unfortunately, there is sporadic information from the occidental part of Mexico, particularly in the crowded city of Guadalajara (1,385,629 habitants, with a surrounding metropolitan area of about 5,268,642 habitants). Since being founded in 1542, the city has undergone innumerable changes in its infrastructure, economy and society. A significant legacy was recently demonstrated regarding medicinal plants, revealing systematized information about their use to treat a variety of illnesses [22]. Based on this background, we aimed to document traditional knowledge about the most common plant species used for medicinal purposes in the five oldest neighborhoods of Guadalajara, Mexico, and to achieve correct taxonomic identification. We used several databases to collect information about the bioactive compounds associated with each species and the potential relation of these bioactive compounds with their empiric therapeutic uses.

## 2. Results and Discussion

From the 137 plant species used in the old neighborhoods of Guadalajara, 5 are only used in one neighborhood, 23 are used in two, 32 are used in three, 63 are used in four and 14 are used in five, namely *Justicia spicigera*, *Schinus molle*, *Matricaria chamomilla*, *Valeriana* sp., *Cucurbita pepo*, *Equisetum arvense*, *Mimosa tenuiflora*, *Salvia officinalis*, *Cinnamomum verum*, *Tilia americana* var. *mexicana*, *Aloysia citrodora*, *Vitis vinifera*, *Zingiber officinale* and *Larrea tridentata* (File S1). Of these, five are native to Mexico, and the rest (9) have been widely cultivated since historical times.

The most diverse neighborhood in terms of botanical families is Mexicaltzingo, with 68 botanical families and 135 species used; the second is San Juan de Dios, with 67 families and 115 species; the third is Analco, with 54 families and 96 species, followed by San Miguel de Mezquitán, with 49 families and 87 species. Finally, El Santuario is the poorest neighborhood, with 22 families and 25 species (File S2).

The local interviews allowed us to generate a database which includes 137 plant species used for medicinal purposes [22] in the old neighborhoods in Guadalajara City. From this information, we found that *J. spicigera*, *M. chamomilla and L. tridentata* were the species with the highest number of medicinal uses (8), followed by *Z. officinale* with five medicinal uses. On the other hand, *C. pepo, M. tenuiflora* and *S. officinalis* had the lowest number of medicinal uses (2) (File S3).

### 2.1. Principal Coordinate Analysis

**Figure 1:**
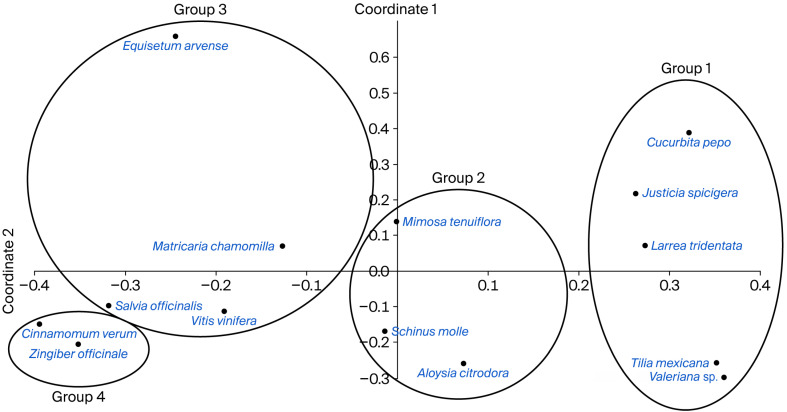
Principal Coordinate Analysis incorporating information about 14 medicinal species used in old neighborhoods of Guadalajara City.

Based on File S4, the PCoA explained 65.88% of the variation accumulated at the first three ordination axes (Table 1) and shaped four groups of species, which are related to the geographical origin of the species: Group 1 is formed by *C. pepo*, *J. spicigera*, *L. tridentata*, *T. americana* var. *mexicana* and *Valeriana* sp. from North America (specifically Mexico). Group 2 contains *M. tenuiflora*, *S. molle* and *A. citrodora* from South and Central America. Group 3 includes *E. arvense*, *M. chamomilla* and *V. vinifera* from Europe. Finally, Group 4 includes *S. officinalis*, *C. verum* and *Z. officinale* from Asia (Figure 1). It is worth noting that non-native species are currently widely cultivated in Mexico and around the world for culinary and medicinal purposes.

### 2.2. Ethnopharmacology

This section is organized alphabetically according to the botanical families of species that are common in the five old neighborhoods of Guadalajara.

#### 2.2.1. Acanthaceae

*Justicia spicigera* Schltdl. is known as “muicle”, “añil de piedra”, “hierba púrpura”, “limanin” or “micle” (File S1a).

This species is used in general blood problems, either to purify, detoxify, compose, increase, or clarify it, and it is also used for blood pressure disorders. In 2010, an outbreak of hemorrhagic dengue fever arose in Guadalajara, and use of this species spread widely as it helped raise the number of platelets in the blood. It is also used in cases of erysipelas, syphilis, tumors, or skin problems that are difficult to cure. This plant is consumed by drinking the reddish violet infusion of its branches and flowers.

A decoction of the leaves or branches, and sometimes the flower, is ingested for ailments related to the digestive system, such as stomachache, diarrhea and dysentery. According to popular beliefs, the decoction of the leaves is ingested to relieve other digestive problems such as fright and gas, and it is also used for sitz baths prepared with the plant’s twigs. The decoction of the leaves is also used as drinking water, acting as an antipyretic, and can treat bad urine and some respiratory conditions such as cough, bronchitis and constipation.

*J. spicigera* is a plant species with great potential as a source of phytochemicals (File S5). Until now, kaempferitrin has been the most studied molecule and is responsible for most biological properties [29]. Ethanolic extract from the branches of this plant showed antibiotic activity against the bacteria *Staphylococcus aureus* and *Bacillus subtilis* [30]. Furthermore, it has an anti-inflammatory effect due to the presence of procumbenoside [31,32].

#### 2.2.2. Anacardiaceae

*Schinus molle* L. is commonly known as “pirul” (File S1b). It contains a red and edible pulp and has antiseptic, antifungal, antiviral, bactericidal, carminative, stimulant and stomachic properties. In addition, it is used as gum emulsion to cure eye diseases such as cataracts and spots on the cornea, and the resin helps to strengthen the gums when chewed. Furthermore, *S. molle* is used to treat urinary tract infections, skin ulcers, gastroduodenal disorders, and ulcers and infections of the respiratory, digestive and genitourinary systems. It is also taken as a diuretic and a digestive tonic to clean the system. It is most frequently used in baths for women during childbirth, but some people believe that the leaves’ extracts could have abortive effects.

With respect to *S. molle*, previous studies have reported a variety of natural phytochemicals (File S5), whereas essential oils of the species are characterized by their antioxidant, antimicrobial [33,34,35,36], cytotoxic and insecticidal properties [36,37,38,39].

#### 2.2.3. Asteraceae

*Matricaria chamomilla* L. is known as “manzanilla” (File S1c). People use its flowers in dried form, which have antispasmodic and anti-inflammatory properties. Chamomile is a reputed anti-inflammatory plant commonly used as a carminative via infusion for the symptomatic treatment of digestive disorders. It is used topically as a softener and antipruritic for dermatological conditions and to treat insect bites and eye irritation.

More than 120 chemical constituents (File S5) in chamomile flower have been identified as secondary metabolites [40,41] with potential pharmacological activity [42]. *M. chamomilla* extracts are dominated by phenolic compounds, including phenolic acids, flavonoids and coumarins. In addition, *M. chamomilla* demonstrates several biological properties such as antioxidant, antibacterial, antifungal, antiparasitic, insecticidal, antidiabetic, anticancer and anti-inflammatory effects. These activities make *M. chamomilla* suitable for application in the medicinal and veterinary fields, in food preservation, phytosanitary control, and as a surfactant and anti-corrosive agent [43]. Moreover, chamomile essential oil, mainly (-)-α-bisabolol, exhibits good activity on promastigote forms of *Leishmania amazonensis* and *L. infantum*, meaning it could be a novel leishmanicidal therapeutic agent [44]. In addition, the gastroprotective effects of apigenin compound found in *M. chamomilla* may be attributed to its anti-inflammatory, antioxidant and anti-apoptotic activities and its ability to enhance the expression of TGF-β1; further experimental research is needed to confirm the activity of apigenin as an anti-ulcer agent [45].

#### 2.2.4. Caprifoliaceae

*Valeriana* sp. is commonly known as “valeriana” or “raiz de gato”. People use the infusion and decoction of underground dried organs such as the rhizome, roots and stolons. These products can be purchased in health food stores or herbal stores where medicinal herbs and natural products are sold; they do not belong to commercial brands but to material collected in the field (according to the sellers themselves). Valerian is used in the symptomatic treatment of neurotonic states, such as minor sleep disorders; some people say that valerian is a common remedy for cases of mental illness. In this study, we could not determine the level of *Valeriana* used in the old neighborhoods of Guadalajara. Some herbaria curators suspected that it was similar to *V. cerathophylla*, but we did not find any studies about it in an extensive search in Web of Science, Wiley, Scopus, Direct Science, Springer, BioOne, Jastor Global Plants, and Jastor. However, we found information about a related Mexican species (*Valeriana edulis* ssp. *procera*), which is distributed in central Mexico and Durango state, and a European species called *V. officinalis*, which has been naturalized worldwide. The major modern and historical uses for valerian are as a sedative and anxiolytic, but it is also used to treat “nervous stomachache”. It significantly improves subjectively recalled sleep quality and shows a favorable adverse effect profile compared with other commonly prescribed sedative hypnotics and anxiolytics. Historically, the herb is used for its sedative/hypnotic and anxiolytic effects to treat other neurological conditions, and it has cardiovascular properties.

Some medical properties of *V. officinalis* have been described in papers, but its main use for the symptomatic treatment of neurotic states such as minor sleep disorders coincides with the uses in Guadalajara’s old neighborhoods [46].

#### 2.2.5. Cucurbitaceae

*Cucurbita pepo* L. is better known as “calabaza”, “pumpkin” or squash in English (File S1e). This species is one of the oldest cultivated species in Mesoamerica, especially in Mexico, together with corn (maiz) and beans, forming a system named “milpa”. The seed is rich in oil, and people use it to treat intestinal parasites and prostate difficulties. The pulp is emollient and used as a sedative for the digestive system; it is also used as an anthelmintic. The male flowers are believed to have a soothing effect when eaten.

The fruits of *C. pepo* are used to cure fatigue and thirst, to purify the blood, and to treat cold and alleviate ache; the seeds are used to treat irritable bladders and prostatic complaints, used as an antiparasitic and taenicide, and are beneficial to the spleen and lungs; they also treat gastritis, burns, enteritis, febrile diseases, headaches and neuralgia. The seeds are diuretic and represent a cure for bronchitis and fever [47]. Finally, the leaves are used to reduce fever, treat nausea and boost the hemoglobin content of the blood. Pumpkin has been considered beneficial to health [48,49] because it contains various biologically active components (File S5).

#### 2.2.6. Equisetaceae

*Equisetum arvense* L. is commonly known as “cola de caballo” (File S1f). This species is used for medicinal purposes due to its diuretic effects and as an auxiliary in maintaining or increasing tissue elasticity. It is also used to increase the body’s defenses and as an astringent. Generally, it is used for its slimming effects and to treat deflated bladder and kidney conditions. Also, there are reports of hemostatic, remineralizing and healing effects. Many people use it for asthenia, convalescence, anemia, fracture consolidation, rheumatism, osteoporosis, cystitis, urethritis, gout, edema and overweight accompanied by fluid retention. It is used topically for wounds; dermal, oral, or corneal ulcerations; blepharitis; conjunctivitis; pharyngitis; dermatitis; erythema; pruritus; and vulvovaginitis.

Previous phytochemical investigations of *E. arvense* have identified a variety of bioactive compounds (File S5), with the major group of constituents being flavonol glycosides and flavonoids (i.e., kaempferol and quercetin derivatives), which confers its antioxidant activity [50]. A strong correlation between cytotoxic activity in cancer cells, antioxidant activity and total phenol content was recorded [51]. It has been documented that the crude extracts and phytochemicals of this plant species exhibited significant antioxidant, antimicrobial, anti-inflammatory, antiulcerogenic, antidiabetic, hepatoprotective and diuretic properties [52]. Also, compounds like phenolic acids, including caffeic acid, ferulic acid and silicic acid, have been linked to various health benefits, including bone health and diuretic effects [53]. It was recently shown that the compounds present in field horsetail extract may protect cells from oxidative stress, inflammation, toxins and metabolic damage [54]. Flavonoids, including quercetin derivatives, particularly their acetylglucosylated forms, exhibit strong antioxidant properties. As a result, they can neutralize reactive oxygen species (ROS) and protect cells from oxidative stress. In addition, hepatoprotective activity is conferred to *E. arvense*, which is attributed to two phenolic petrosins, onitin and onitin-9-O-glucoside, along with four flavonoids, apigenin, luteolin, kaempferol-3-O-glucoside and quercetin-3-O-glucoside [55].

#### 2.2.7. Fabaceae

*Mimosa tenuiflora* Poir. is known as “tepezcohuite” (File S1g). People use this species to treat odontalgia, inflammation of external organs, injuries, fever, menstrual cramps, headaches, hypertension, bronchitis and cough. Also, *M. tenuiflora* is part of Mexican folk medicine, being used to treat wounds, parasites and gastrointestinal problems.

After a series of catastrophes in Mexico during the 1980s (the 1982 Chichonal volcano eruption in Chiapas; the 1984 gas explosion in San Juan Ixhuatepec, State of Mexico; the 1985 Mexico City earthquake; and the 1986 plane crash between Mexico City and Toluca, State of Mexico), the use of tepezcohuite bark against skin wounds and burns became popular [56]. Unfortunately, this Mimosa species, like others, contains alkaloids, which may limit its pharmacological development as an OTC (“over the counter”) drug due to national and international legal requirements.

The phytochemistry of *M. tenuiflora* has attracted considerable interest (File S5), mainly due to the presence of indole alkaloids and tannins (proanthocyanidins) [57]. All of these secondary metabolites and others not included in this study are responsible for its medicinal properties [58]. However, in the old neighborhoods of Guadalajara, only the bark of the trunk is used as a healing agent for burns [59], and it is effective in curing venous leg ulcers. However, in a vast review of the plant species’ uses, taxonomy and anatomy, it was stated that “although in Mexico there is a rich herbal tradition and deep-rooted traditional medicine, no references have been found on the medicinal use of “tepezcohuite” by pre-Hispanic indigenous groups” [56]. In the same line, any data or record of the medicinal use of “tepezcohuite” was found by indigenous groups located in or near the current distribution area of this species in Mexico, such as the Zoques (in the N-NW of Chiapas and E-NE of Oaxaca), the Mixes and Popolocas (to the E, NE and SE of Oaxaca), the Huaves (in the E region of Tehuantepec and to the S of the Isthmus of Tehuantepec, Oaxaca) and the Zapotecs (in Tehuantepec and in the center of the state of Oaxaca). The only antecedent of the medicinal use of this plant is the reference on the label of a botanical specimen from the state of Chiapas [58]. Although it has been determined that its medicinal use is not of pre-Hispanic origin, it is important to note that this species is part of the folk medicine of Mexico; its medicinal potential has been validated from pharmacological and cytotoxic analyses of its bark. However, additional studies are required before its sale as a medicine can be authorized [56].

#### 2.2.8. Lamiaceae

*Salvia officinalis* L is commonly known as “salvia” (“common sage” in English) (File S1h). People use the dried leaves, whole or fragmented. Historically, sage is known as the “salvation plant”, originating from the old Latin word “salvarem”, which means save or cure [60]. Sage enjoys a reputation as a panacea since it has antispasmodic (spasmolytic) properties; it is also used to treat digestive disorders, as an oral hygiene rinse, and for the treatment of small wounds. The leaf extract is used as a toner, cleanser, anti-dandruff agent, antioxidant, antiperspirant, deodorant, skin protectant, astringent, antimicrobial agent, skin conditioner and soother.

There are at least nine known pharmacological effects of *S. officinalis*, including anti-inflammatory, antioxidant, hypoglycemic, antiviral, antianxiety, antifungal, memory improving, and anticancer activities, along with effects on Alzheimer’s disease [61]. The bioactive compounds of this species (File S5) are shown in its polyphenol profile and have been extensively investigated and reviewed [60]. However, sage has more beneficial effects, and with the range of new extraction techniques available nowadays, new components with innovative therapeutic effects are being discovered, especially in the context of neurodegenerative diseases and various carcinomas. In particular, chemical and pharmacological investigations of sage revealed ursolic acid as the main component involved in its anti-inflammatory activity [62].

#### 2.2.9. Lauraceae

*Cinnamomum verum* J. Presl. is better known as “canela” (File S1i) or cinnamon and is one of the most consumed spices. According to the Indian System of Medicine (Ayurveda), its medicinal use has been documented for over 6000 years [63].

People use canela as a symptomatic treatment for digestive disorders, functional fatigue and loss of appetite, but it has contraindications in pregnancy and ulcers. Cinnamon is also used to clean intoxicated blood and to treat coughs, “the scare” and insomnia. Some people believe it has antipyretic properties. It is also used in digestive disorders, such as colitis, stomach pain and coldness, as well as to “cut the stomach” after eating highly acidic foods and to treat dysentery, diarrhea, vomit and bile. People use cinnamon as a spice, but it is also used to cure some gynecological disorders. Additionally, the bark of the plant is used to eliminate a condition that is described as a pain in the pit of the stomach caused by not eating and that can spread throughout the body.

The most important cinnamon oils in world trade are those from *C. zeylanicum*, *C*. *cassia* and *C. camphora*, and the essential oil composition of cinnamon varies depending on the geographical origin of the spices and the processing conditions [64]. Nevertheless, though numerous species of this genus are sold as cinnamon, the inner dried bark of *C. verum* J. Presl is considered true cinnamon [65]. In fact, cinnamon is used as a spice in daily life without any side effects. Several reports have allocated numerous properties of cinnamon in the forms of bark, essential oils, bark powder, phenolic compounds, flavonoids and isolated components (File S5), and each of these properties plays a key role in the advancement of human health. Antioxidant and antimicrobial activities may occur through direct action on oxidants or microbes, whereas anti-inflammatory, anticancer, and antidiabetic activities occur indirectly via receptor-mediated mechanisms [63].

#### 2.2.10. Tiliaceae

*Tilia americana* var. *mexicana* (Schltdl.) Hardin is commonly known as “tila estrella” or “lime blossom” in English (File S1j).

The infusion of lime blossom is popularly known; it is used to calm the nerves but also often used to treat heart disease and blood pressure. It has also been said to relieve menstrual colic. In general, it is recommended to drink the decoction of the flower to treat all of these conditions. It is a plant species native to Mexico, historically used as a sedative; the few existing pharmacological studies of this plant confirm the effectiveness of its medicinal application.

Aerial tissues of *T. americana* var. *mexicana* are used in traditional Mexican medicine to treat several central nervous system-related illnesses, such as anxiety, headache and insomnia [66]. Infusions of the flowers and fruits are used to treat colon spasms, menstrual irregularities, rheumatism and hypertension [67,68]. Significantly, different experimental models have suggested that organic extracts have anticonvulsant, neuroprotective and hepatoprotective effects and antioxidant and cytotoxic activities [69,70,71]. The protective effects of *T. americana* var. *mexicana* involve antioxidant activity, which may lie beneath its traditional medicinal effects and biomedical properties and suggests that it possesses neuro and hepatoprotective activity. This demonstrates its potential in preventing age-related deterioration of the brain or liver and as applications for neuro and hepatopathies [71]. In particular, the sedative activities [69,72] of inflorescence and leaf methanolic extracts are attributed to several chemical constituents (File S5).

#### 2.2.11. Verbenaceae

*Aloysia citrodora* Paláu is commonly known as “cedrón” (File S1k) or lemon verbena; it is a perennial plant that grows widely in South and Central America and in various parts of the Middle East, including Jordan.

Different extracts of *A. citriodora* have been shown to possess strong antimicrobial, anticonvulsant, anticolitis, antioxidant, cytoprotective, anticonceptive, anti-inflammatory, estrogenic and anticancer effects and can weakly inhibit the hyperpropulsive movement of the small intestine [73]. These uses could be related to the phytochemical constituents contained in the species (File S5).

In Mexico, people traditionally use leaves as infusion in the symptomatic treatment of various digestive disorders and neurotonic states such as minor sleep disorders. They are also used for their essential oils’ properties, such as antiseptic, antispasmodic, carminative, detoxifying, digestive, febrifuge, sedative, stomach soothing and liver–biliary stimulating effects. This plant is considered medicinal as it can resolve disorders of the digestive system such as diarrhea, biliary colic, vomiting and gas, but its most frequent medicinal use is for stomach pain. For the treatment of these conditions, the leaves are boiled and administered orally.

#### 2.2.12. Vitaceae

*Vitis vinifera* L. is better known as “uva” (fruit) or “parra” (plant) (File S1l).

People use dried red vine leaf in the symptomatic treatment of functional disorders of cutaneous capillary fragility (ecchymosis, petechiae, venous insufficiency and hemorrhoidal symptoms), and it is used topically to treat ocular discomfort. The fruit extract is also used as a venotonic, vasoprotective, astringent and diuretic for the treatment of varicose veins, hemorrhoids, phlebitis, edema, capillary fragility and diarrhea.

*V. vinifera* and its bioactive compounds (File S5) have several pharmacological activities, such as antioxidative, anti-inflammatory and antimicrobial activities, as well as in vitro activity against several cancer cell lines and hepatoprotective and cardioprotective effects [74]. An important review has shown that this valuable plant exhibits beneficial effects in different components of metabolic syndrome, including dyslipidemia, diabetes, hypertension and obesity. It also exhibits cardioprotective and hepatoprotective activities [75]. Phenolic compounds are the most important active constituents of grapes [76]; grape seed polyphenols have an antitumor effect [77], and they should be studied in more detail for use as cancer chemopreventive and/or anticarcinogenic agents.

#### 2.2.13. Zingiberaceae

*Zingiber officinale* Roscoe is commonly known as “jengibre” or ginger (File S1m). The rhizome has anti-inflammatory activity and no side effects due to its non-toxic nature; it is used to prevent motion sickness, to treat digestive disorders and as a spasmolytic, antiemetic, positive cardiac inotrope and stimulant of intestinal peristalsis and salivary and gastric secretions. In herbal medicine, ginger is recommended for a very wide range of digestive problems, such as tiredness or lack of energy, motion sickness and nausea, especially during pregnancy.

*Z. officinale* has a long history of medicinal use as an anti-inflammatory agent for musculoskeletal diseases in Ayurvedic and Chinese medicine [78]. Moreover, several of its chemical constituents (File S5), including gingerols, shogaols, paradols and zingerone, have demonstrated anti-inflammatory actions in vitro, inhibiting leukotriene synthesis, the activity of cyclooxygenase enzymes (COX-1 and COX-2), the production of interleukins (Il-1 and Il-12), and tumor necrosis factor alpha in activated macrophages [79,80].

#### 2.2.14. Zygophyllaceae

*Larrea tridentata* (DC.) Coville is commonly known as “gobernadora” (File S1m). In particular, *L. tridentata* is a perennial shrub used in traditional medicine in northern Mexico and the Southern United States to treat infertility, rheumatism, arthritis, colds, diarrhea, skin problems, pain, inflammation and excess body weight.

Scientific research has revealed the beneficial effects of *L. tridentata* as an antioxidant and antitumor, neuroprotective, regenerative, antibacterial, antiviral, antifungal, anthelmintic, antiprotozoal and insecticidal agent, although reports indicate that some compounds in *L. tridentata* may be hepatotoxic and nephrotoxic [81]. *L. tridentata* is a species rich in bioactive compounds (File S5).

Guadalajara is the most populated city with the greatest historical relevance in the Central Region of Jalisco State. Since the creation of its first neighborhoods, various macro projects have been developed over time in the historic center or first square [82]. It is remarkable that traditional neighborhoods and their inhabitants still preserve some ancestral traditions, among which different plant species have long been used in traditional medicine as herbal preparations for their calming effects and sedative action and to counter depression [22]. Similar findings have been reported in the area of Varanasi, India, which has been settled for millennia and now represents a large urban environment; despite dense urbanization, medicinal plants still play a key role in the health care of the local population [83]. Furthermore, plants commonly used as traditional medicines in rural areas can still be found in the city and are collected and used by the local population.

However, even though knowledge about the use of medicinal plants persists in Guadalajara’s oldest neighborhoods, this traditional knowledge is at risk of being lost due to factors such as major economic and social transformations and gentrification, which displace original inhabitants and cause them to leave their places of residence, taking their ethnobotanical knowledge with them.

One of the oldest neighborhoods in Guadalajara where gentrification has been recorded to a greater degree is El Santuario, where a constant decline in its population has been occurring for several decades, to the point where almost 25% of its homes are unoccupied and a high percentage are businesses [84]. This neighborhood is significant as it is currently recognized as the place where patent medicines of dubious origin are sold, which is why they maintain prices below those commercially available in pharmacies. Additionally, a botanical garden was built in front of the Guadalajara Civil Hospital in 1793, located in this neighborhood, with the intention of cultivating plants related to empirical medicine [85]. Despite the renovations made in recent years, this botanical garden faces troubling conditions: accumulating garbage, overflowing containers, unpleasant odors, the presence of homeless people and insecurity. Years ago, attempts were made to recover the space due to its appropriation by homeless people and street vendors, which motivated the Guadalajara City Council to seek balanced solutions to restore the site’s original purpose [86].

Many clinical trials have pointed out evidence of the efficacy of herbal medicinal products and have suggested the right of this practice to occupy a steady place in modern drug therapy. Most people believe that phytotherapy is generally regarded as relatively low risk. Even though side effects and interactions cannot be excluded, a special feature of herbal medicines is that they usually have a lower rate of side effects than synthetic drugs. Many medicinal plants have been used as teas, powdered medicines or alcoholic extracts (tinctures and drops) for decades or even centuries. Due to intensive research and modern technology, extracts can now be concentrated and marketed as capsules or tablets which contain the active ingredients of the plant [4].

## 3. Materials and Methods

### 3.1. Study Area

**Figure 2:**
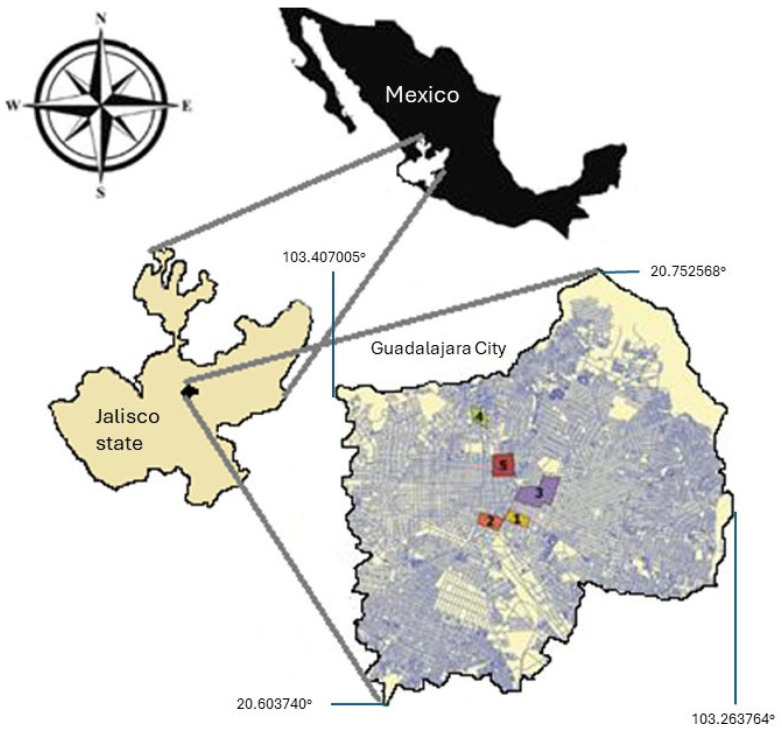
Geographical location of Guadalajara City. Yellow 1 = Analco; Orange 2 = Mexicaltzingo; Purple 3 = San Juan de Dios; Green 4 = San Miguel de Mezquitán; Red 5 = El Santuario.

The city of Guadalajara is situated in the Atemajac Valley and is part of the province of the Transmexican Neovolcanic Belt between 103°22′ N and 20°40′ W (Figure 2), with an average altitude of 1583 m a.s.l. Most of the territory constitutes low hills, and the highest point is called “Cerro del Cuatro”. It has a semi-warm climate with medium humidity, summer rains and mostly dry winters ((A)Ca(w1)(w)(e)g) according to the Köppen’s climate classification system modified by [87], with an average annual temperature above 18 °C. The city of Guadalajara is the capital of the Jalisco State, and it is the most populous area with the greatest historical relevance in the Central Region of Jalisco State.

### 3.2. Field Work and Interviews

In 2023, we made a series of visits to interview local people in the old neighborhoods of Guadalajara. A previous exploratory visit was made in June 2022 to locate houses growing any medicinal plant species, and a second visit was made to learn about the owners of those houses and to make appointments for later interviews regarding those medicinal plants and others as well as their uses. All plant species were recorded to create an ethnobotanical database containing all information.

Based on ethnobotanical studies previously carried out in the same old neighborhoods [22], we focused our interviews on people older than 40 years because this is the age group that holds more traditional knowledge about medicinal plants; each interviewee had lived continuously in the area for at least 30 years. To maximize data collection and allow the interviewees to speak freely, the interviews were semi-structured [88]. Four questions were asked to all people interviewed: (1) What is the name of the plant? (2) What are the uses of this plant that you know of? (3) What parts of the plant are utilized (root, stem, leaf, inflorescence, flower or fruit)? (4) How is the product/utensil prepared (raw, cooked, roasted or ground)? (based on [89,90]). Informed consent was obtained from each of the informants before the interviews were conducted according to the International Society of Ethnobiology Code of Ethics 2006 [89].

### 3.3. Systematic and Literature Review

According to a previous study [22], in the old neighborhoods of Guadalajara, there are 137 plant species with medicinal use; of these, 14 of them are commonly used in the five old neighborhoods (File S1). Since these species have a higher number of uses, we performed a systematic review of the origin and modes of use for different illnesses, as well as the chemical components of each species.

The taxonomic identification of plants was performed based on different monographies and corroborated with herbarium specimens from the digital herbarium of Missouri Botanical Garden [91] and Red Nacional de Herbarios del Noroeste de México. All collected and identified specimens were stored at the IBUG herbarium of the Instituto de Botánica de la Universidad de Guadalajara, also named “Herbario Luz María Villarreal de Puga”. The correct nomenclatural species names can be found in the IPNI [92].

### 3.4. Multivariate Analysis

To explore the relationships among the 14 species commonly used in the old neighborhoods of Guadalajara, a Principal Coordinate Analysis (PCoA), was performed using PAST software version 4.11 (Natural History Museum, University of Oslo, Oslo, Norway) [93], the information obtained from literature reviews (including geographical origin, biochemical profile or chemical components) and the information obtained from the interviews (the ways the plants are used for different illnesses and their effects) (File S5). PCoA is an ordination technique that works well with relatively homogeneous data sets [94] and is especially useful in ecological analysis with non-quantitative descriptors or with data sets containing many zeros. This method allows one to position objects (plant species in our case) in a space of reduced dimensionality while preserving their distance relationships as well as possible [95]. PCoA is equivalent to Principal Component Analysis, but the latter assumes linear combinations of the original descriptors (linear relationships), while PCoA is a function of the original variables but is mediated through the similarity or distance function that has been used [95].

## 4. Conclusions

Eight of the fourteen plant species commonly used as medicine in the old neighborhoods of Guadalajara are of American origin, five are native to Mexico, and the remaining one comes from Europe and Asia. These fourteen species can be found either cultivated or growing spontaneously in fields, which makes them accessible and practical therapeutic resources. They are often present in home gardens, along roadsides, within the traditional milpa system, in abandoned croplands or in areas of disturbed vegetation near communities. In addition, they are commercialized in regional markets, which contributes to the persistence of their use.

*J. spicigera*, *L. tridentata* and *M. chamomilla* are the medicinal plant species with the highest number of uses, with the first two being native to Mexico and the latter being native to Europe. *Valeriana* sp., *C. pepo*, *M. tenuiflora*, *A. citrodora* and *V. vinifera* are used for medicinal purposes for specific diseases (to treat the nervous system, intestinal parasites, burns, pain in the chest and cancer, respectively); the first two are Mexican species, the following two are South American species and the last is a European species.

Although the medicinal use of these species by local inhabitants is based on empirical knowledge, all of them—except *Valeriana* sp.—have been the subject of various phytochemical and biological studies, providing solid support for their use and efficacy. Despite the widespread use of *Valeriana* sp., it could not be identified precisely, and no studies in the scientific literature were found regarding its bioactive compounds.

However, despite the continued use of medicinal plants in Guadalajara’s older neighborhoods, the gentrification process that has taken place in recent decades has caused many of these neighborhoods’ residents to leave their homes, taking their traditional knowledge with them. This is especially the case in the El Santuario neighborhood, which is located near Guadalajara’s Historic Center.

## Figures and Tables

**Table 1:** The explained variance for each of the first three ordination axes.

Explained Variance:	Axis 1	Axis 2	Axis 3
Extracted	42.99%	12.76%	10.13%
Cumulative	29.861%	55.75%	65.88%

## Data Availability

Collected specimens and herbarium vouchers are deposited at Ethnobotanical Herbario Luz María Villarreal de Puga del Instituto de Botánica de la Universidad de Guadalajara (IBUG). The collection number belongs to R.E.M.G.

## Supplementary Materials

The following supporting information can be downloaded at: https://www.mdpi.com/article/10.3390/plants14172788/s1, File S1. Medicinal plant species commonly used in the five old neighborhoods of Guadalajara, Jalisco, Mexico. File S2. Medicinal plant species and families used in each old neighborhood of Guadalajara, Jalisco, Mexico. File S3. Traditional medicinal uses of the 14 plant species in the five old neighborhoods of Guadalajara, Jalisco, Mexico. File S4. Characteristics of the medicinal plant species used in the five old neighborhoods of Guadalajara, Jalisco, Mexico. In all cases, 0 = absence and 1 = presence, except in geographical origin, where 1 = Mexico, 2 = South or Central America, 3 = Europe and 4 = Asia. File S5. Biomolecules identified by different authors in the 14 medicinal plant species commonly used in the old neighborhoods of Guadalajara, Jalisco, Mexico. References [69,73,96,97,98,99,100,101,102,103,104,105,106,107,108,109,110,111,112,113,114,115,116,117,118,119,120,121,122,123,124,125,126,127,128,129,130,131,132,133,134,135,136,137,138,139,140,141,142,143,144,145,146,147,148,149,150,151,152,153,154,155,156] are cited in the supplementary materials.
