# Supplementary material for: Ethnobotany in a Modern City: The Persistence in the Use of Medicinal Plants in Guadalajara, Mexico
Source: Plants (Basel). 2025 Sep 5;14(17):2788. doi: 10.3390/plants14172788 (PMC12430341; doi:10.3390/plants14172788)
Supplement: Supplementary file 1 [file plants-14-02788-s001.zip › File S1 Species pictures.pdf]

File S1

File S1. Medicinal plant species commonly used in the five old neighborhoods of Guadalajara, Jalisco, Mexico

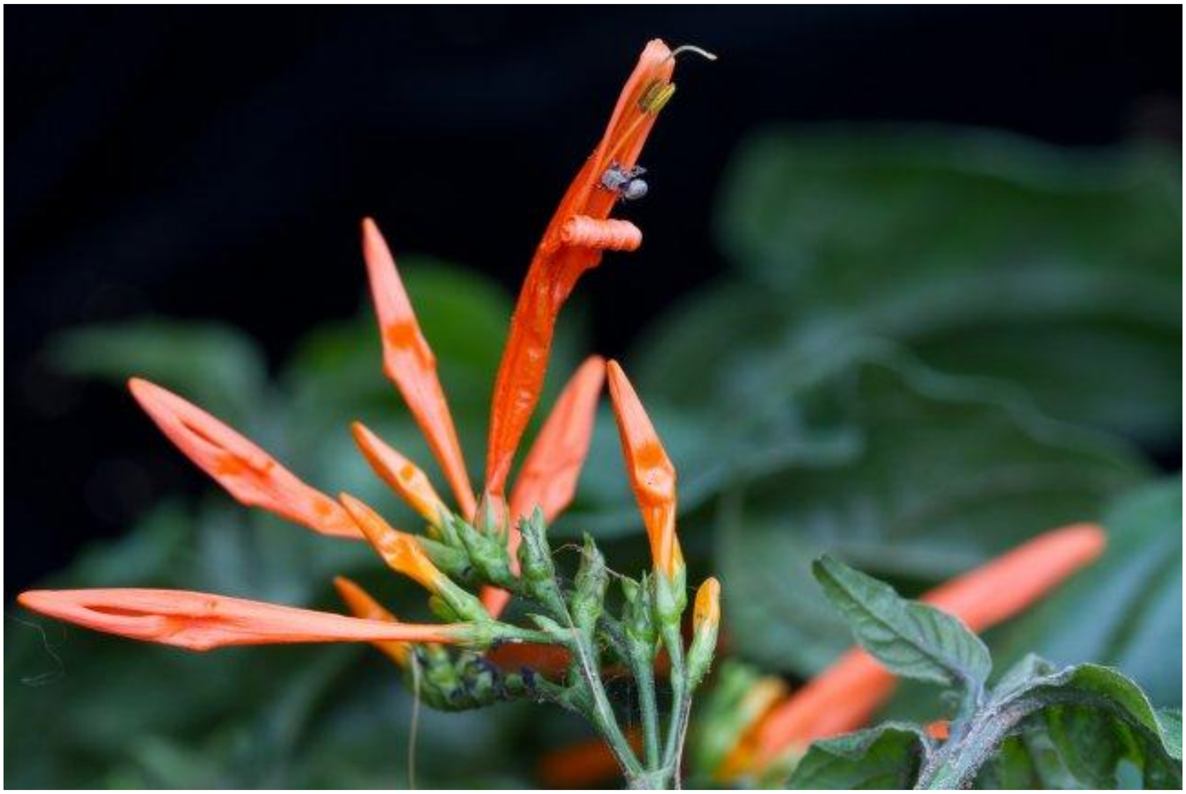

a) ***Justicia spicigera*** Schltdl.

Tropicos.org. Missouri Botanical Garden. <http://www.tropicos.org/Image/100167033> 05 Aug 2025

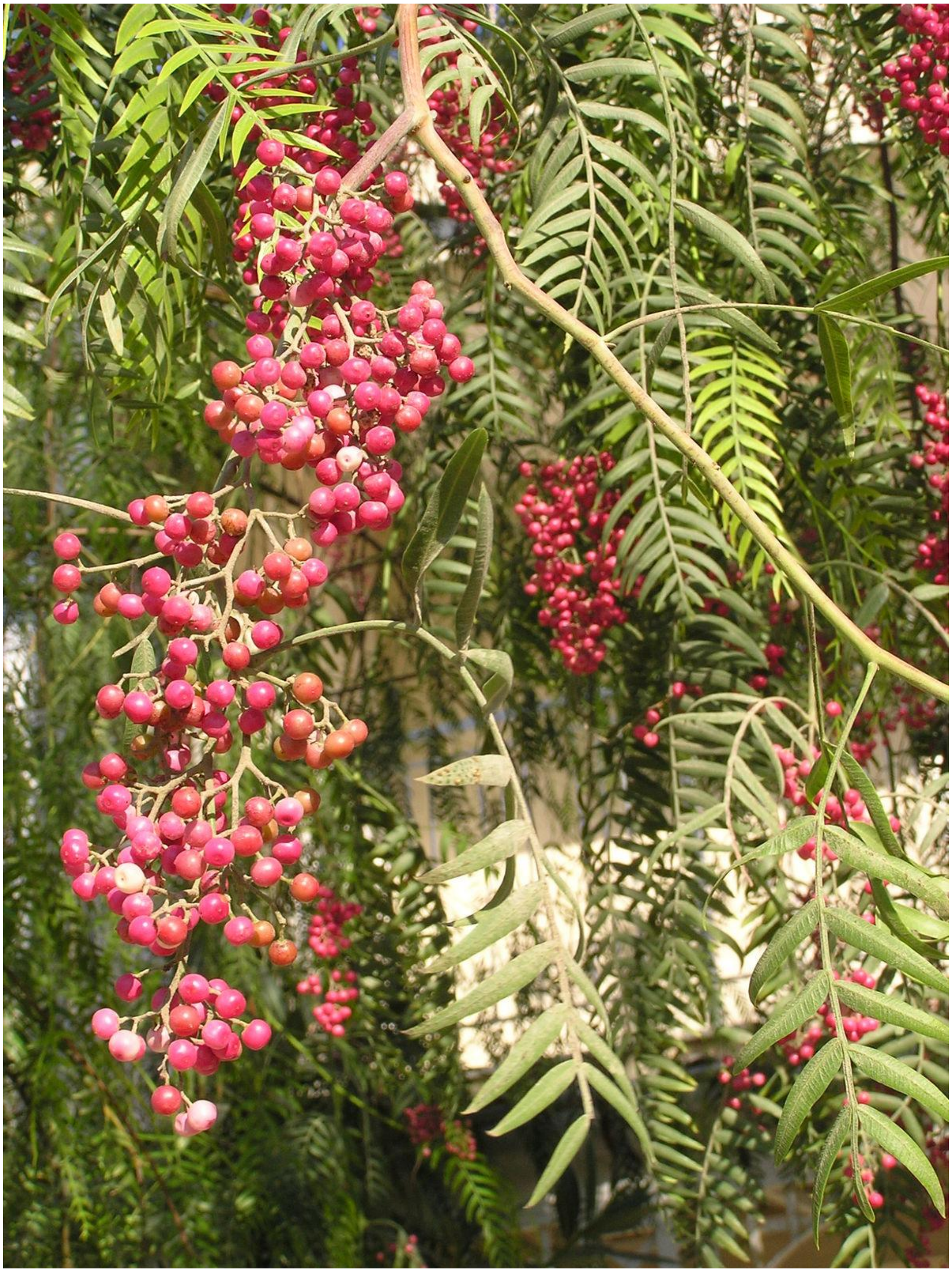

b) ***Schinus molle*** L.

Tropicos.org. Missouri Botanical Garden. <http://www.tropicos.org/Image/100120417> 05 Aug 2025

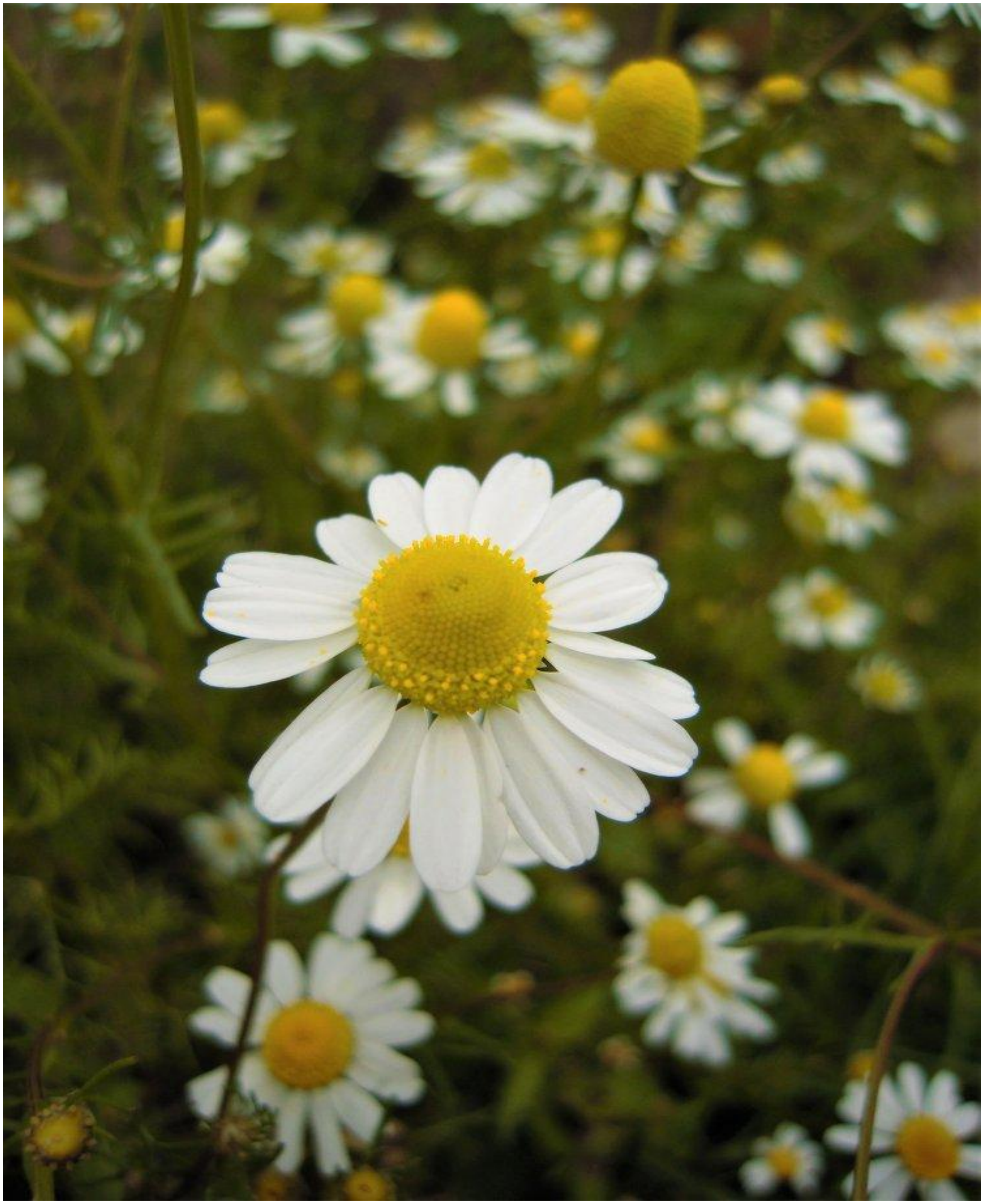

c) ***Matricaria chamomilla*** L

Tropicos.org. Missouri Botanical Garden. <http://www.tropicos.org/Image/100774359> 05 Aug 2025

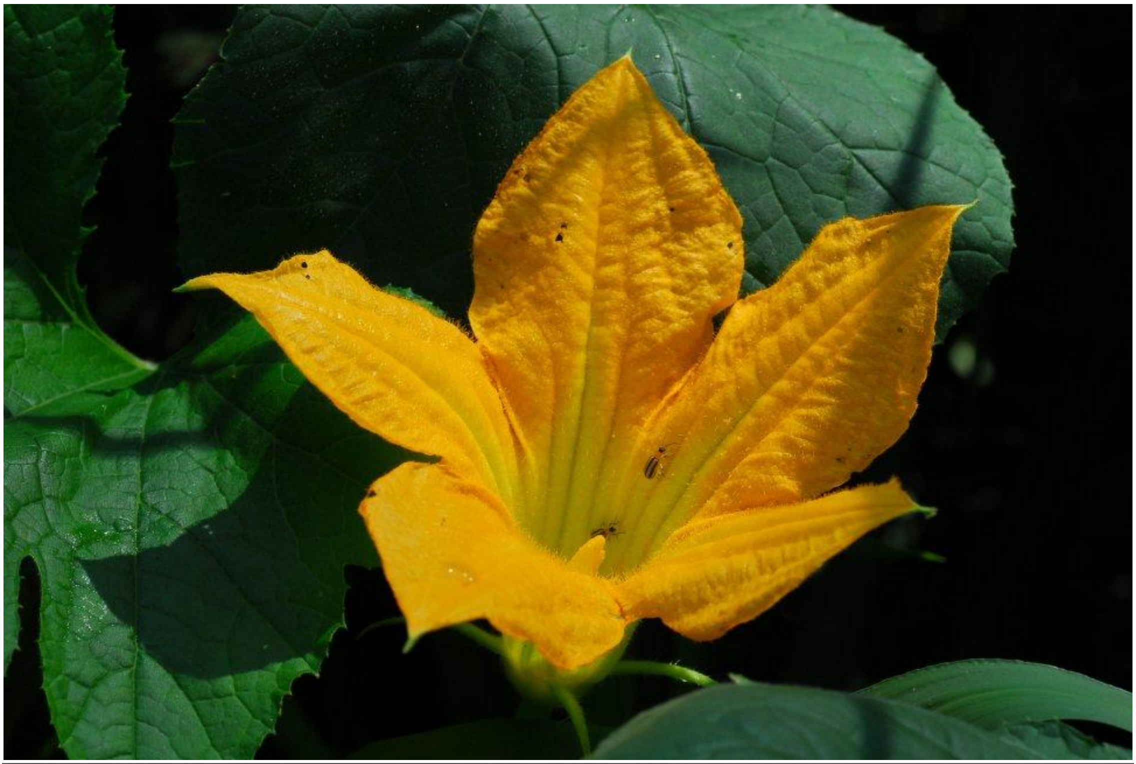

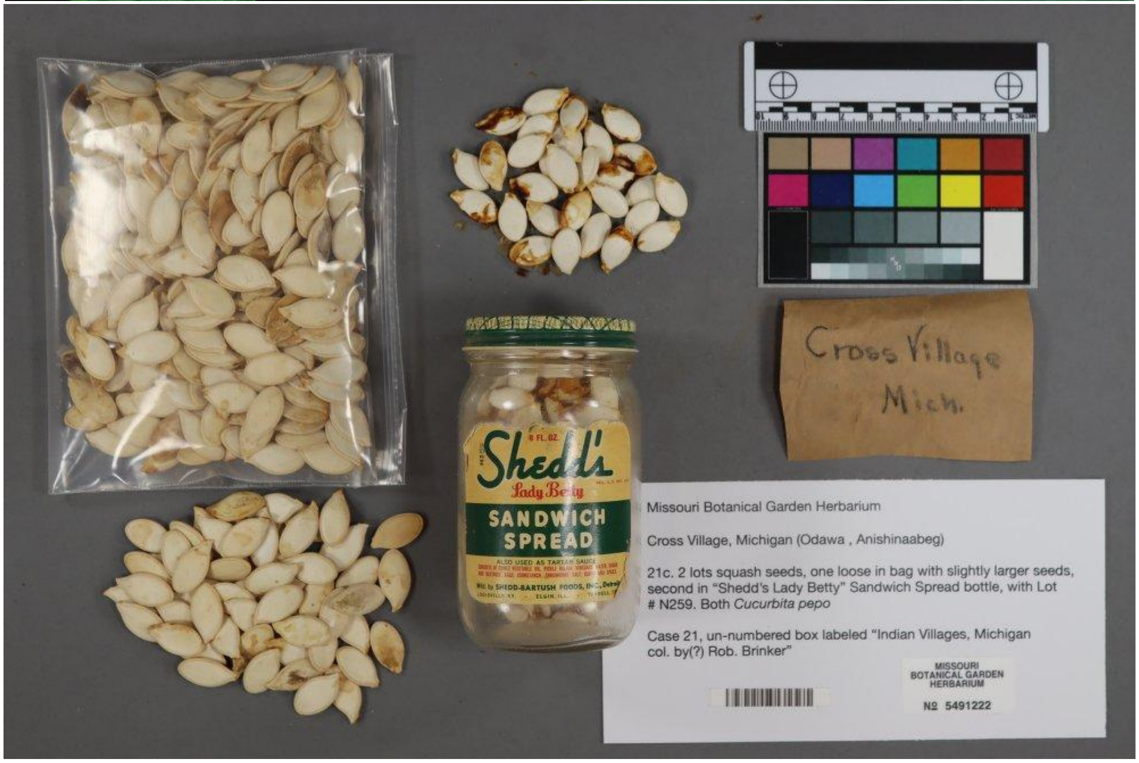

d) ***Cucurbita pepo*** L.

Tropicos.org. Missouri Botanical Garden. <http://www.tropicos.org/Image/100208388> 05 Aug 2025

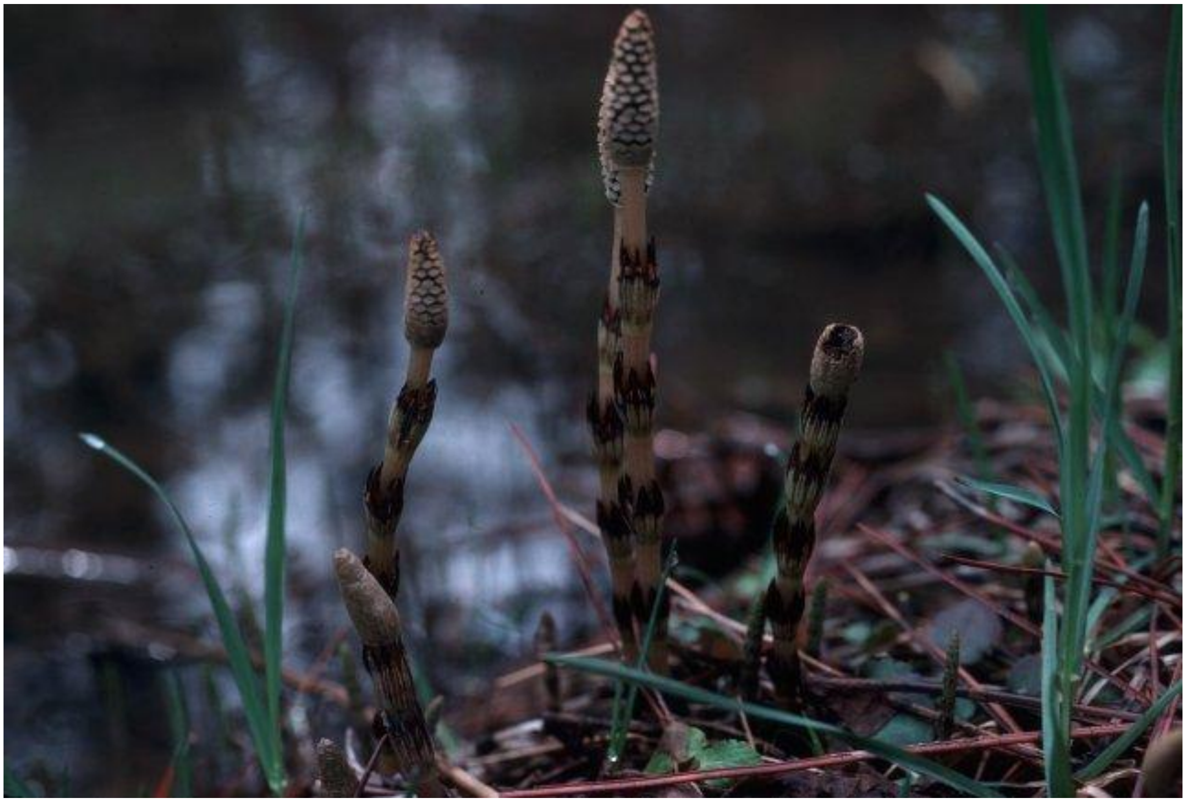

e) ***Equisetum arvense*** L.

Tropicos.org. Missouri Botanical Garden. <http://www.tropicos.org/Image/100783604> 05 Aug 2025

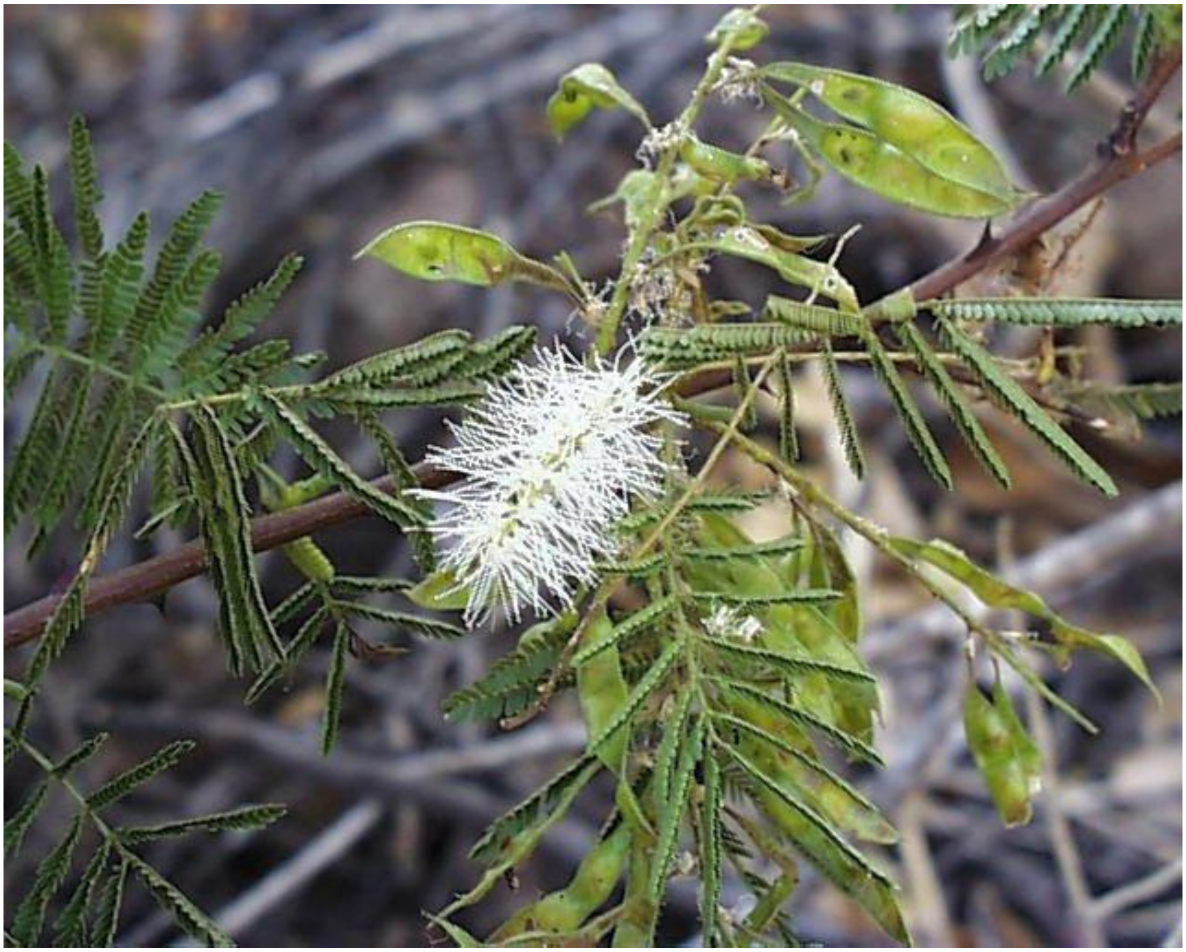

f) *Mimosa tenuiflora* Poir.

Tropicos.org. Missouri Botanical Garden. <http://www.tropicos.org/Image/69017> 05 Aug 2025

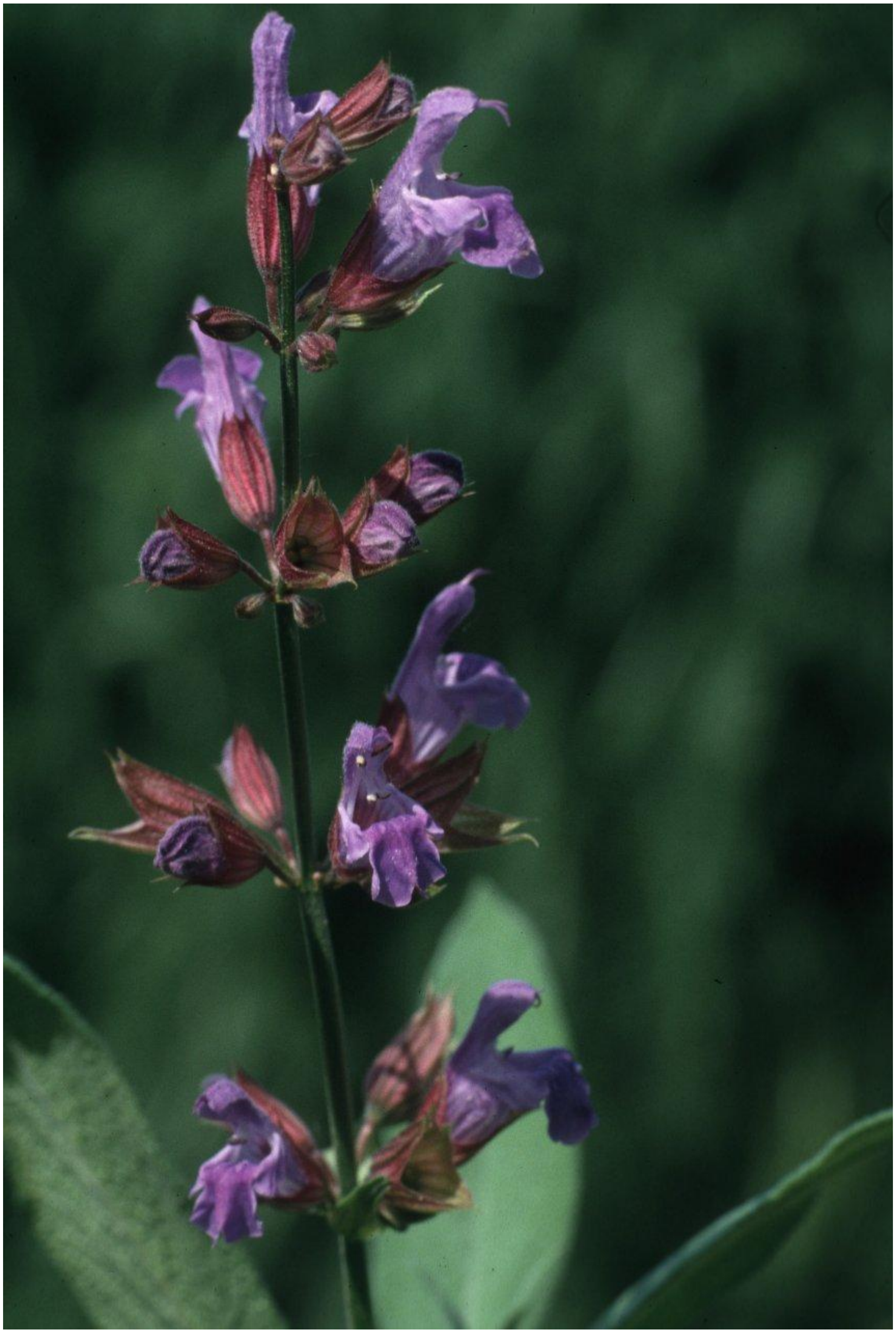

g)* *Salvia officinalis L.

Tropicos.org. Missouri Botanical Garden. <http://www.tropicos.org/Image/100828114> 05 Aug 2025

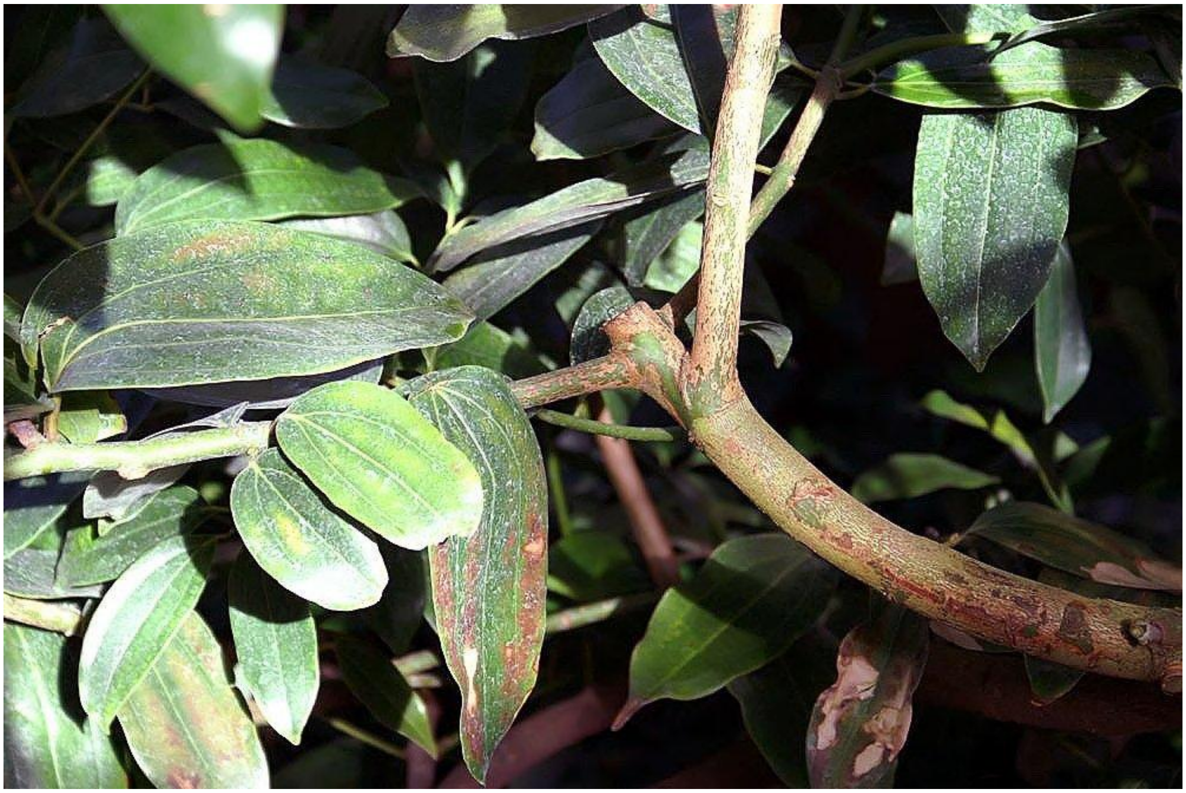

h) *Cinnamomum verum* J. Presl.

Tropicos.org. Missouri Botanical Garden. <http://www.tropicos.org/Image/100110001> 05 Aug 2025

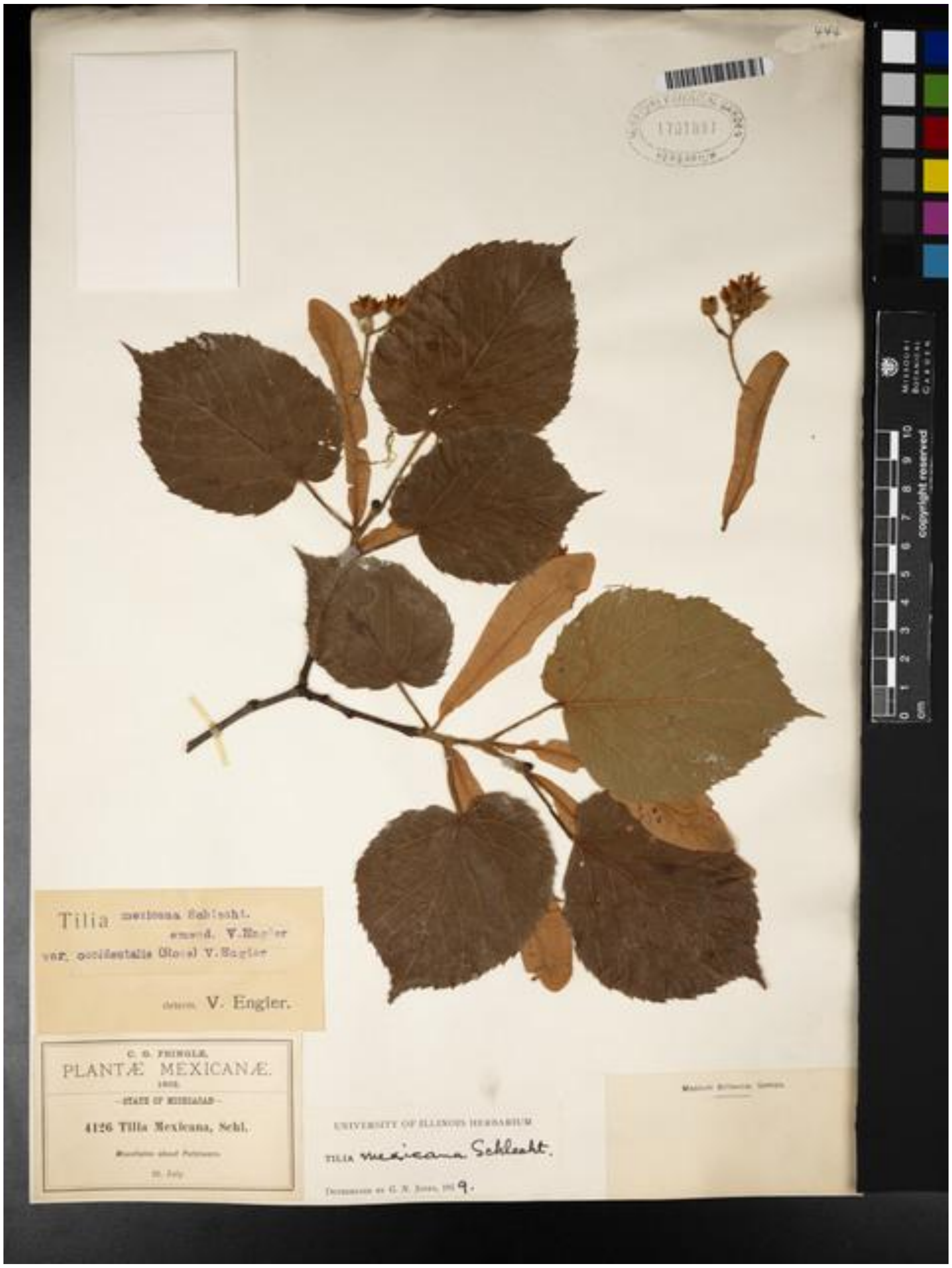

i) ***Tilia americana*** **var.** ***mexicana*** (Schltdl.) Hardin

Tropicos.org. Missouri Botanical Garden. <http://www.tropicos.org/Image/102867023> 06 Aug 2025

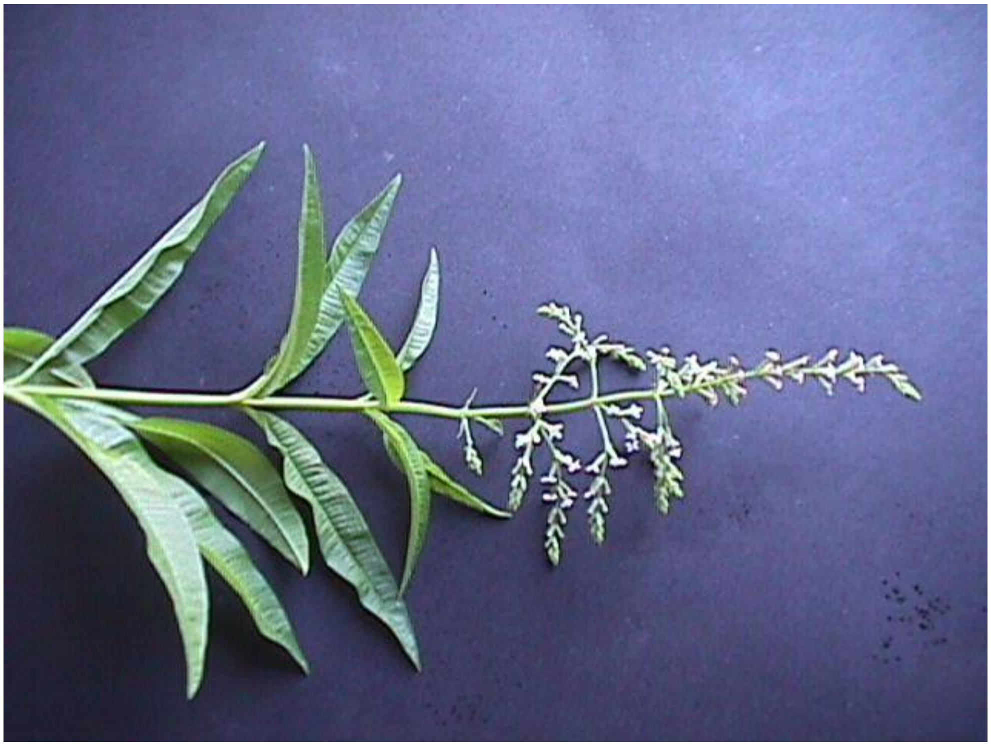

j) ***Aloysia citrodora*** Paláu

<https://www.plantasyhongos.es/herbarium/htm/Aloysia_citrodora.htm> 06 Aug 2025

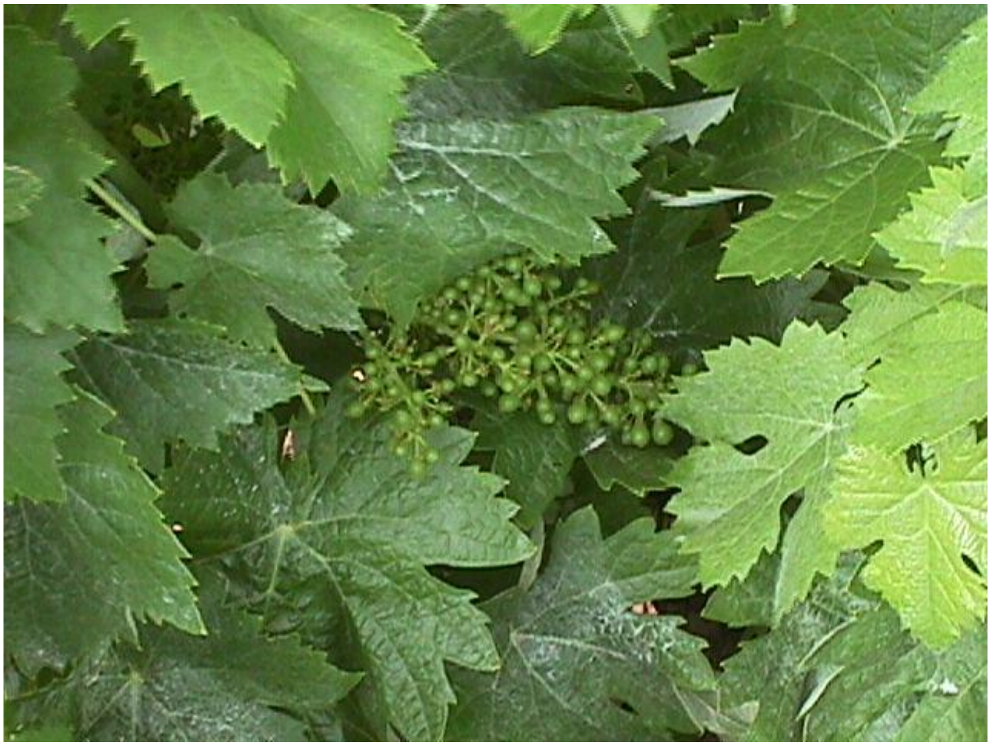

k) *Vitis vinifera* L.

<https://www.plantasyhongos.es/herbarium/htm/Vitis_vinifera.htm> 05 Aug 2025

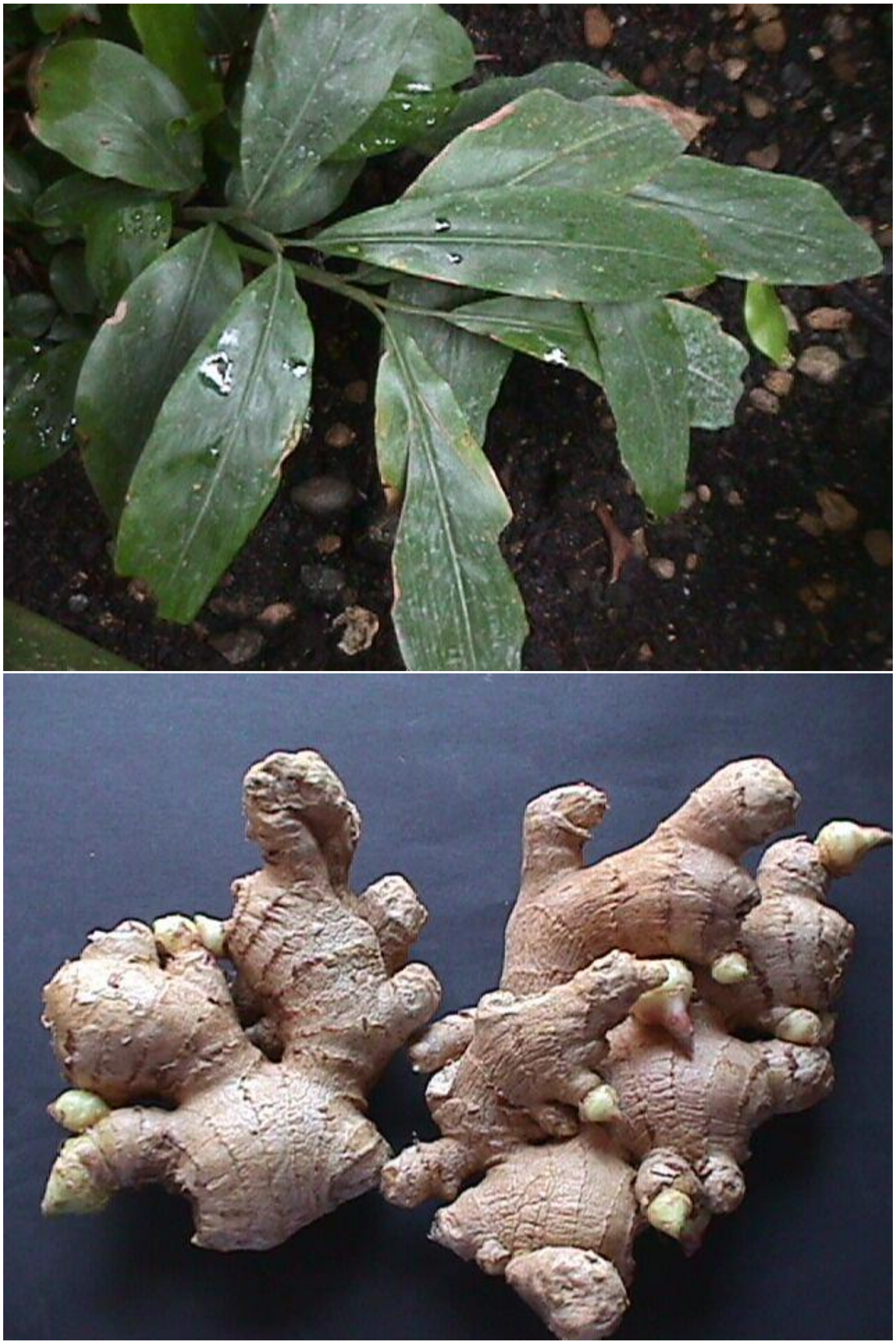

l) ***Zingiber officinale*** Roscoe

<https://www.plantasyhongos.es/herbarium/htm/Zingiber_officinale.htm> 06 Aug 2025

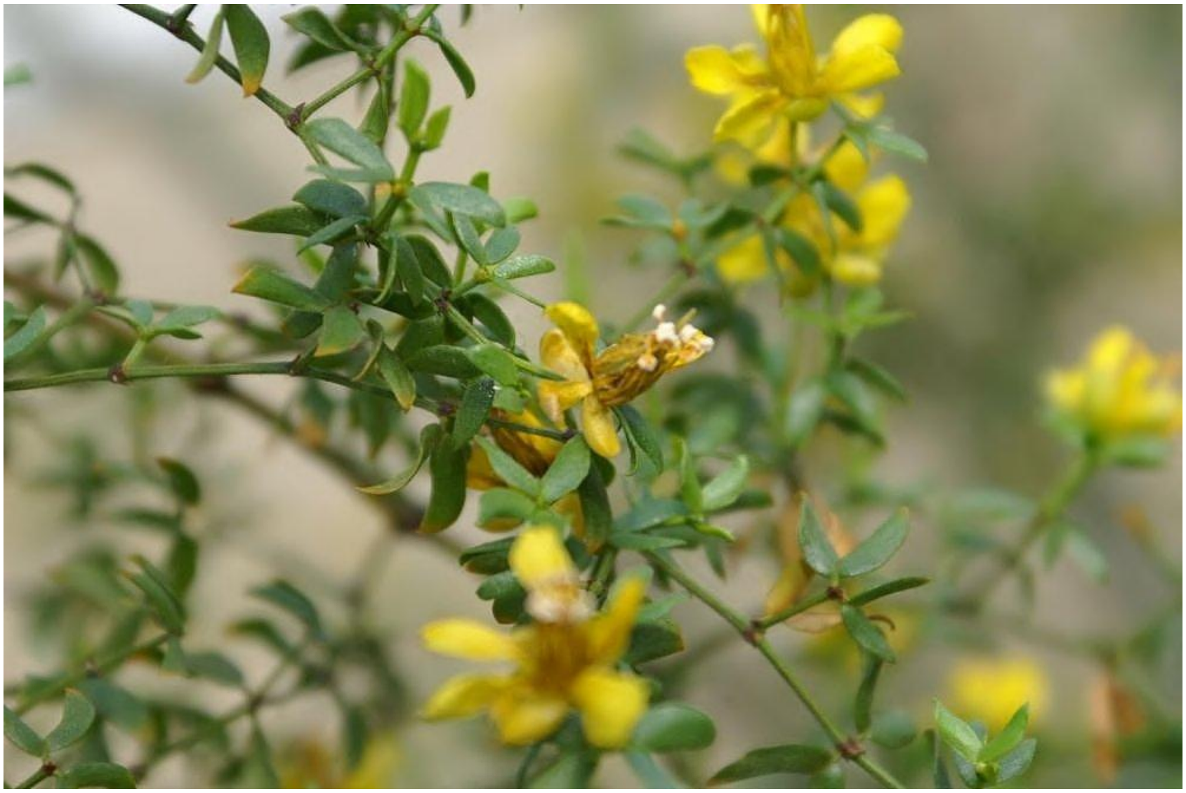

m) ***Larrea tridentata*** (DC.) Coville

Tropicos.org. Missouri Botanical Garden. <http://www.tropicos.org/Image/100115201> 06 Aug 2025
