# Supplementary material for: Ethnobotany in a Modern City: The Persistence in the Use of Medicinal Plants in Guadalajara, Mexico
Source: Plants (Basel). 2025 Sep 5;14(17):2788. doi: 10.3390/plants14172788 (PMC12430341; doi:10.3390/plants14172788)
Supplement: Supplementary file 1 [file plants-14-02788-s001.zip › File S2 Species and families per neighborhood.pdf]

File S2. Medicinal plant species and families used in each old neighborhood of Guadalajara, Jalisco, Mexico.

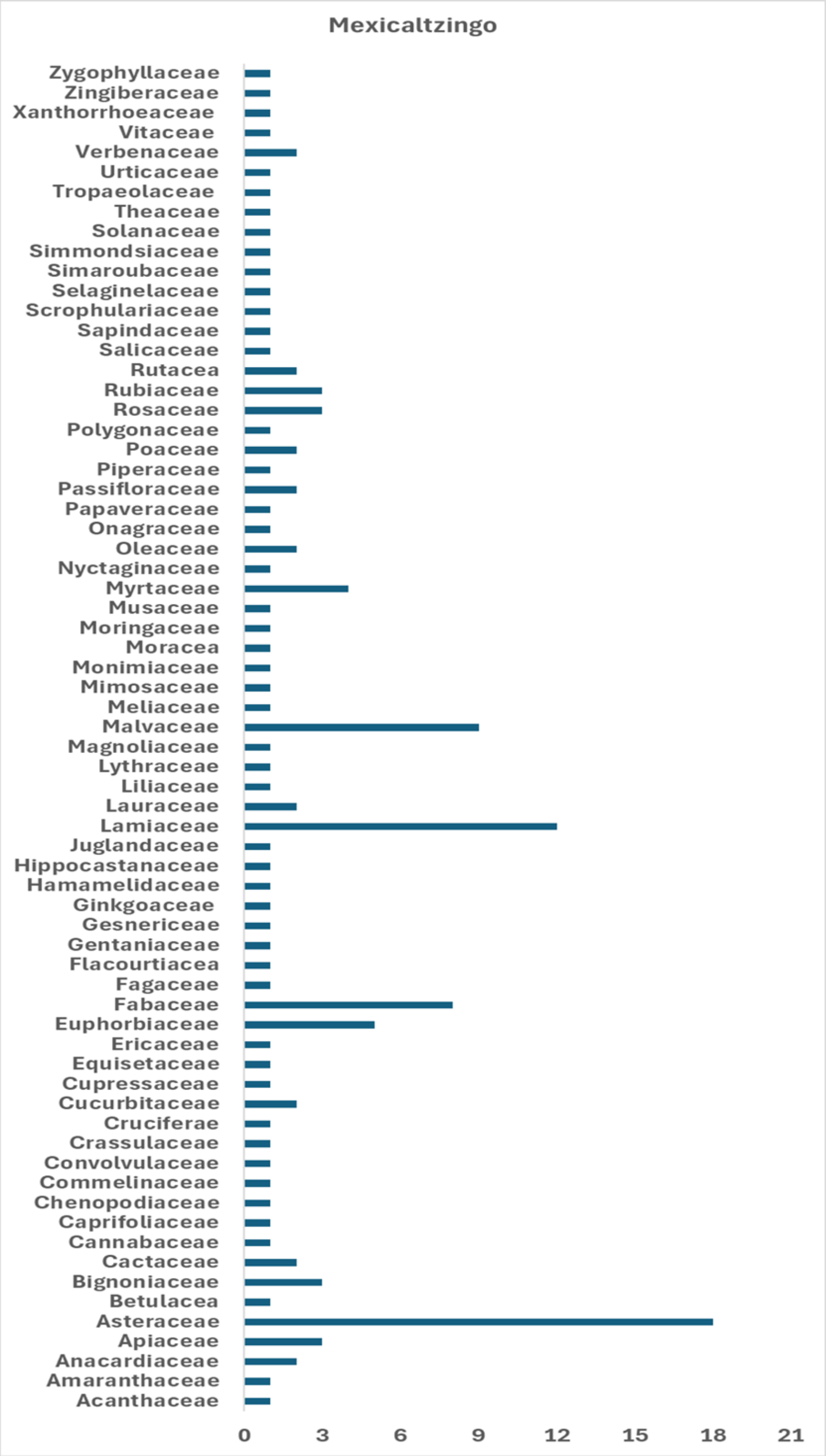

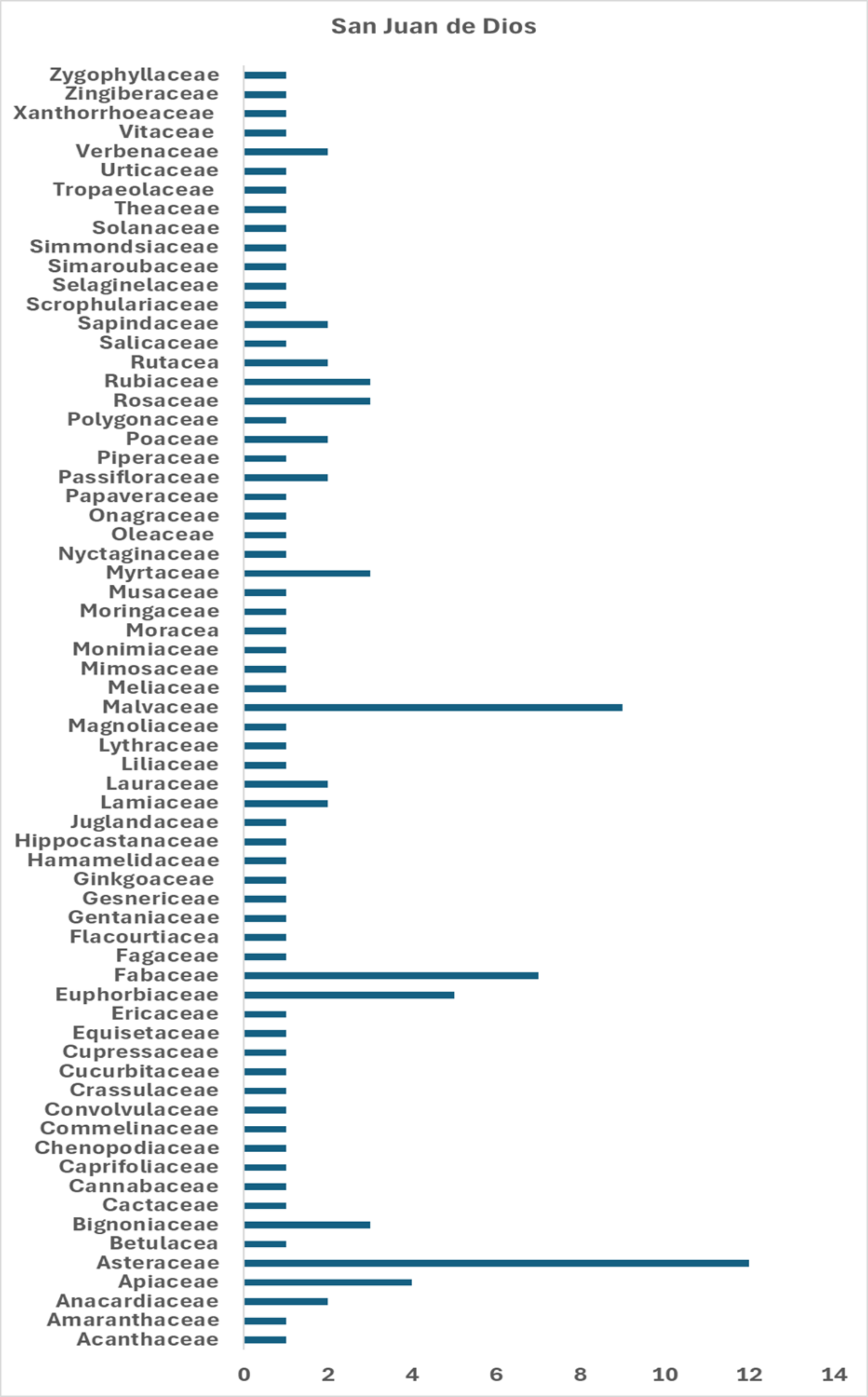

San Juan de Dios
Zygophyllaceae
Zingiberaceae
Xanthorrhoeaceae
Vitaceae
Verbenaceae
Urticaceae
Tropaeolaceae
Theaceae
Solanaceae
Simmondsiaceae
Simaroubaceae
Selaginelaceae
Scrophulariaceae
Sapindaceae
Salicaceae
Rutacea
Rubiaceae
Rosaceae
Polygonaceae
Poaceae
Piperaceae
Passifloraceae
Papaveraceae
Onagraceae
Oleaceae
Nyctaginaceae
Myrtaceae
Musaceae
Moringaceae
Moracea
Monimiaceae
Mimosaceae
Meliaceae
Malvaceae
Magnoliaceae
Lythraceae
Liliaceae
Lauraceae
Lamiaceae
Juglandaceae
Hippocastanaceae
Hamamelidaceae
Ginkgoaceae
Gesnericeae
Gentaniaceae
Flacourtiacea
Fagaceae
Fabaceae
Euphorbiaceae
Ericaceae
Equisetaceae
Cupressaceae
Cucurbitaceae
Crassulaceae
Convolvulaceae
Commelinaceae
Chenopodiaceae
Caprifoliaceae
Cannabaceae
Cactaceae
Bignoniaceae
Betulacea
Asteraceae
Apiaceae
Anacardiaceae
Amaranthaceae
Acanthaceae
0
2
4
6
8
10
12
14

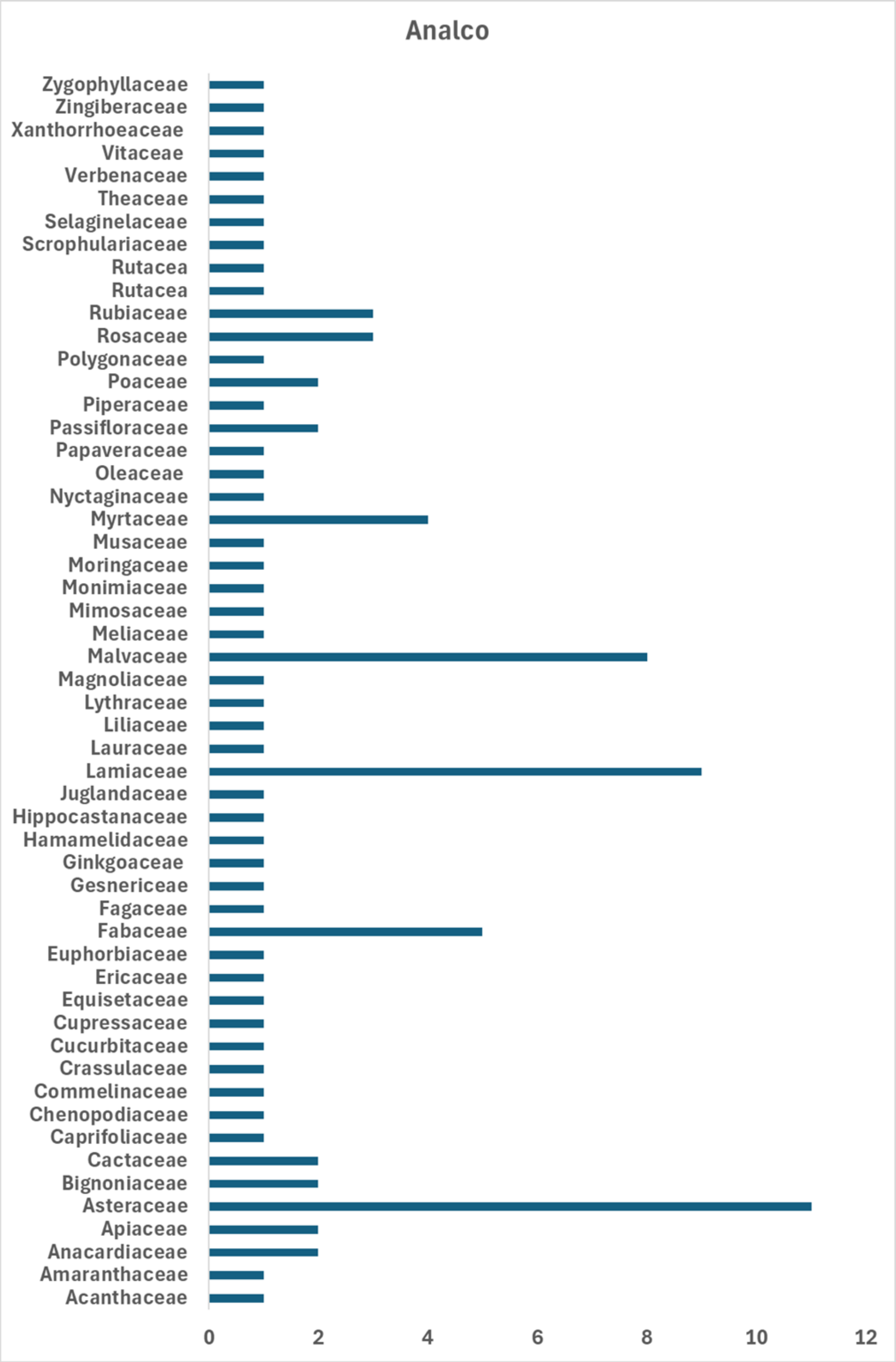
Analco
Zygophyllaceae
Zingiberaceae
Xanthorrhoeaceae
Vitaceae
Verbenaceae
Theaceae
Selaginelaceae
Scrophulariaceae
Rutacea
Rutacea
Rubiaceae
Rosaceae
Polygonaceae
Poaceae
Piperaceae
Passifloraceae
Papaveraceae
Oleaceae
Nyctaginaceae
Myrtaceae
Musaceae
Moringaceae
Monimiaceae
Mimosaceae
Meliaceae
Malvaceae
Magnoliaceae
Lythraceae
Liliaceae
Lauraceae
Lamiaceae
Juglandaceae
Hippocastanaceae
Hamamelidaceae
Ginkgoaceae
Gesnericeae
Fagaceae
Fabaceae
Euphorbiaceae
Ericaceae
Equisetaceae
Cupressaceae
Cucurbitaceae
Crassulaceae
Commelinaceae
Chenopodiaceae
Caprifoliaceae
Cactaceae
Bignoniaceae
Asteraceae
Apiaceae
Anacardiaceae
Amaranthaceae
Acanthaceae
0
2
4
6
8
10
12

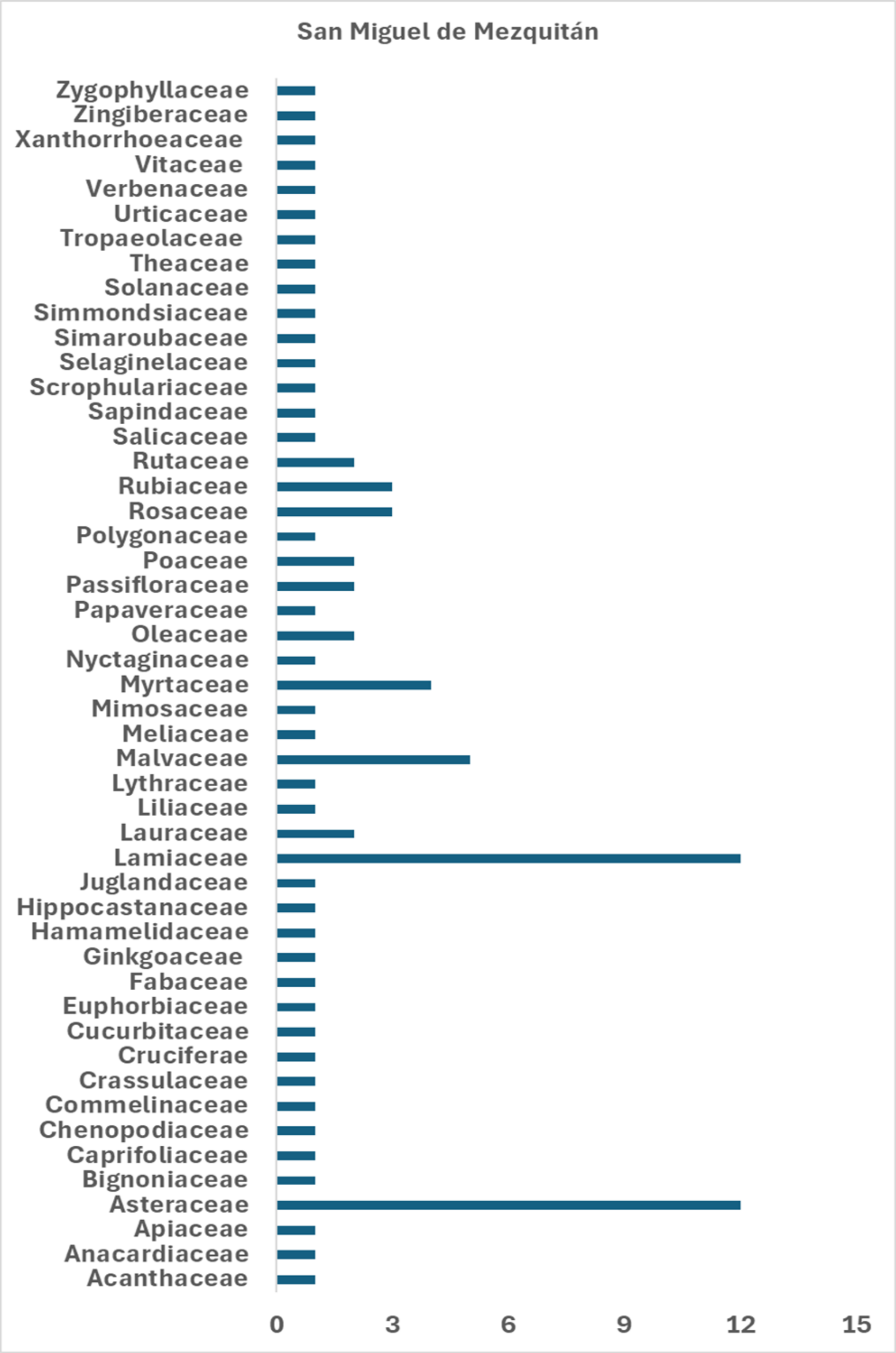
San Miguel de Mezquitán
Zygophyllaceae
Zingiberaceae
Xanthorrhoeaceae
Vitaceae
Verbenaceae
Urticaceae
Tropaeolaceae
Theaceae
Solanaceae
Simmondsiaceae
Simaroubaceae
Selaginelaceae
Scrophulariaceae
Sapindaceae
Salicaceae
Rutaceae
Rubiaceae
Rosaceae
Polygonaceae
Poaceae
Passifloraceae
Papaveraceae
Oleaceae
Nyctaginaceae
Myrtaceae
Mimosaceae
Meliaceae
Malvaceae
Lythraceae
Liliaceae
Lauraceae
Lamiaceae
Juglandaceae
Hippocastanaceae
Hamamelidaceae
Ginkgoaceae
Fabaceae
Euphorbiaceae
Cucurbitaceae
Cruciferae
Crassulaceae
Commelinaceae
Chenopodiaceae
Caprifoliaceae
Bignoniaceae
Asteraceae
Apiaceae
Anacardiaceae
Acanthaceae
0
3
6
9
12
15

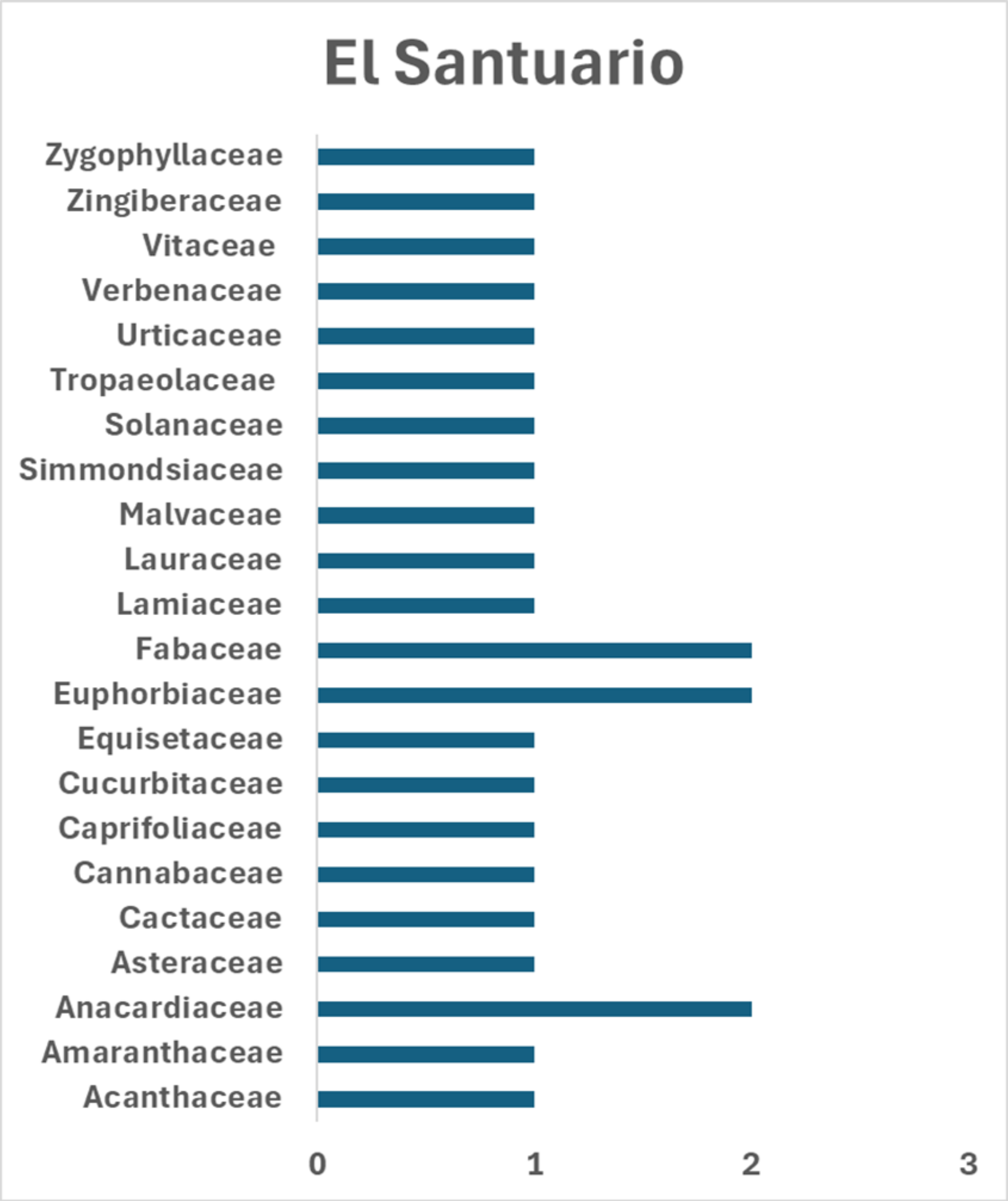
El Santuario
Zygophyllaceae
Zingiberaceae
Vitaceae
Verbenaceae
Urticaceae
Tropaeolaceae
Solanaceae
Simmondsiaceae
Malvaceae
Lauraceae
Lamiaceae
Fabaceae
Euphorbiaceae
Equisetaceae
Cucurbitaceae
Caprifoliaceae
Cannabaceae
Cactaceae
Asteraceae
Anacardiaceae
Amaranthaceae
Acanthaceae
0
1
2
3
