# Supplementary material for: Ethnobotany in a Modern City: The Persistence in the Use of Medicinal Plants in Guadalajara, Mexico
Source: Plants (Basel). 2025 Sep 5;14(17):2788. doi: 10.3390/plants14172788 (PMC12430341; doi:10.3390/plants14172788)
Supplement: Supplementary file 1 [file plants-14-02788-s001.zip › File S3 Traditional medicinal use of the 14 plant species.pdf]

File S3

File S3. Traditional medicinal use of the 14 plant species commonly used in the five old neighborhoods of Guadalajara, Jalisco, Mexico. Number in parentheses denotes the number of diseases for which the species is used.

Species	Analco	San Juan de Dios	Mexicaltzingo	Mezquitán	El Santuario
Justicia spicigera Schltdl.	(7) Dysentery, anemia, menstrual flow control, lowers fever, colic, menopaus, purifies the blood	(7) Dysentery, anemia, menstrual flow control, lowers fever, colic, menopause, purifies the blood	(8) Dysentery, anemia, menstrual flow control, lowers fever, colic, menopause, purifies the blood, flu.	(2) purifies the blood, influenza	(1) purifies the blood
Schinus molle L.	(2) Urinary, genito-urinary tract diseases	(4) Urinary, genito-urinary tract diseases nerves, eyes	(2) Urinary, genito-urinary tract diseases	(2) Urinary, genito-urinary tract diseases	(1) Urinary tract
Matricaria chamomilla L.	(6) Arthritis, menstrual cramps, conjunctivitis, catarrh, varicose veins, dyspepsia	(6) Arthritis, menstrual cramps, conjunctivitis, catarrh, varicose veins, dyspepsia	(6) Arthritis, menstrual cramps, conjunctivitis, catarrh, varicose veins, dyspepsia	(6) Arthritis, menstrual cramps, conjunctivitis, catarrh, varicose veins, dyspepsia	(4) Arthritis, menstrual cramps, conjunctivitis, catarrh, varicose veins, dyspepsia gastritis nervosa, diarrhea
Valeriana sp.	(3) Nerves, insomnia, madness	(3) Nerves, insomnia, madness	(3) Nerves, insomnia, madness	(1) Nerves	(1) Nerves
Cucurbita pepo L.	(1) Intestinal parasites	(2) Intestinal parasites, anticarcinogenic	(1) Intestinal parasites	(1) Intestinal parasites	(1) Intestinal parasites

Equisetum arvense L.	(3) slimming, deflates bladder, kidney	(3) slimming, deflates bladder, kidney	(3) slimming, deflates bladder, kidney	(4) slimming, deflates bladder, kidney, parasites	(2) deflates bladder, kidney
Mimosa tenuiflora Poir.	(1) Healing burns	(1) Healing burns	(1) Healing burns	(1) Healing burns	(2) Healing burns, acne
Salvia officinalis L.	(2) Bad digestion, prevent cancer	(2) Bad digestion, prevent cancer	(2) Bad digestion, prevent cancer	(2) Bad digestion, prevent cancer	(2) Bad digestion, prevent cancer
Cinnamomum verum J. Presl.	(3) Altered nerves, indigestion, losing weight	(3) Altered nerves, indigestion, losing weight	(3) Altered nerves, indigestion, losing weight	/1) lose weight	(3) Indigestion, cholesterol, heart disease
Tilia americana var. *mexicana* (Schltdl.) Hardin	(2) depression, cough	(3) depression, cough, influenza	(3) depression, cough, influenza	(2) depression, cough	(3) depression, cough, anxiety
Aloysia citrodora Paláu	(1) air pain in chest	(1) air pain in chest	(1) air pain in chest	(1) air pain in chest	(3) air pain in chest, lowers fever, colic
Vitis vinifera L.	(1) Cancer	(1) Cancer	(1) Cancer	(1) Cancer	(3) Cancer, nerves, cough
Zingiber officinale Roscoe	(2) Constipation, expel bile production	(2) Constipation, expel bile production	(2) Constipation, expel bile production	(2) Constipation, expel bile production	(4) Anti-inflammatory, expel bile production, nausea, digestion
Larrea tridentata (DC.) Coville	(5) Arthritis, rheumatism, kidneys, sterility, purgative	(5) Arthritis, rheumatism, kidneys, sterility, purgative	(5) Arthritis, rheumatism, kidneys, sterility, purgative	(2) Kidneys, sterility	(5) Infertility, sexual impotence, kidney stones, bladder, prostate
