# Supplementary material for: Ethnobotany in a Modern City: The Persistence in the Use of Medicinal Plants in Guadalajara, Mexico
Source: Plants (Basel). 2025 Sep 5;14(17):2788. doi: 10.3390/plants14172788 (PMC12430341; doi:10.3390/plants14172788)
Supplement: Supplementary file 1 [file plants-14-02788-s001.zip › File S4 Characteristics of the plant species used.pdf]

File S4.

File S4. Characteristics of the plant species used in the old neighborhoods of Guadalajara. In all cases 0=absence 1=Presence, except in Geographical origin where 1= Mexico, 2= South America, 3=Europe and 4= Asia.

	Justicia spicigera	***Schinus molle***	***Matricaria chamomilla***	***Valeriana* sp.**	***Cucurbita pepo***	***Equisetum arvense***	***Mimosa tenuiflora***	***Salvia officinalis***	***Cinnamomum verum***	***Tilia mexicana***	***Aloysia citrodora***	***Vitis vinifera***	***Zingiber officinale***	***Larrea tridentata***
Geographical Origin	1	2	3	1	1	4	2	4	4	1	2	3	4	1
Blood problems	1	0	0	0	0	1	1	0	1	0	0	0	1	0
Artherial pressure	1	0	1	0	0	0	1	0	1	1	0	0	1	0
Stomachache	1	0	1	0	0	0	0	0	1	0	0	0	1	0
Cough, bronchitis	1	0	0	0	0	1	1	0	1	0	0	0	1	1
Skin problems	1	0	1	0	1	1	0	0	0	0	0	0	0	1
Healing	0	0	1	0	1	1	1	1	0	0	0	0	0	0
Diuretic	0	0	0	0	1	1	0	0	0	0	0	0	0	1
Sedative	0	0	1	1	0	0	0	0	0	1	1	0	0	0
Mestrualache	1	0	0	0	0	1	1	0	0	1	0	0	0	0
Bad urine	1	0	0	0	0	0	0	0	0	0	0	0	0	1
Antiinflamatory	0	0	1	0	0	0	1	1	1	0	0	1	1	1
Eye infections	0	1	1	0	1	1	0	0	1	0	0	0	0	0
Insomnia	0	0	0	1	0	0	0	0	0	1	1	0	0	0
Kidney problems	0	0	0	0	0	1	0	0	0	0	0	0	0	1
Gums	0	1	0	0	1	1	1	0	0	0	0	0	0	0
Antioxidative	1	0	0	0	1	1	0	1	1	0	0	1	1	0
Hypoglicemic	0	0	0	0	0	0	0	1	1	1	0	1	1	0
Anticeptic	0	1	0	0	0	0	0	1	1	0	0	0	1	1
Antimicrobial	1	1	1	0	0	1	1	1	1	0	1	1	1	1
Antiviral	0	1	0	0	0	0	1	0	1	0	0	1	1	1
Antifungic	0	1	1	0	0	0	0	0	1	0	0	1	0	1
Essential oil	1	1	1	0	0	0	0	1	1	0	1	1	1	0
Flavonoids	1	0	1	0	1	1	1	1	0	1	1	1	0	1
Tanines	1	0	1	0	1	1	0	0	0	0	0	1	0	0
Phenols	0	0	0	0	0	1	0	1	1	0	0	1	0	0

Terpenes	0	1	1	1	0	1	1	1	1	0	1	0	1	1
Alkaloids	0	0	0	1	1	1	1	1	0	0	0	0	0	1
