# Supplementary material for: Ethnobotany in a Modern City: The Persistence in the Use of Medicinal Plants in Guadalajara, Mexico
Source: Plants (Basel). 2025 Sep 5;14(17):2788. doi: 10.3390/plants14172788 (PMC12430341; doi:10.3390/plants14172788)
Supplement: Supplementary file 1 [file plants-14-02788-s001.zip › File S5 Biomolecules.pdf]

File S5. Biomolecules identified by different authors in the 14 medicinal plant species commonly used in the old neighborhoods of Guadalajara, Jalisco, Mexico.

Plant species	Phytochemical profile	Reference
Justicia spicigera Schltdl.	Alkaloids,steroids,tannins,terpenoids,lignans and flavonoids (e.g., naringenin)	[96]
	4-methyl-3-pentanal, methyl 2-hydroxy-2-methyl-butanoate, 3,4-epoxy-2-hexanone, 2-hydroxy-2-methylbutyric acid, ethyl 2-hexenoate, 3-thiophene-acetic acid, phthalic acid and butyl 2-ethylbutyl ester	[97]
	Peonidin 3,5-diglucoside, malvidin 3,5-diglucoside, petunidin 3,5-diglucoside	[98]
	Simple carbohydrates; pectins; glycoproteins; phenolic compounds (glycosides of kaempferol) and anthocyanins (cyanidin 3-O-glucoside and cyanidin 3,5-di glucoside); phenolic compounds (kaempferitrin and kaempferol trirhamnoside)	[99]
	Eucalyptol, phytol and azulene. Flavonoids, sterols, and terpenes	[100, 101]
	Procumbenoside B (i.e., 4-O-beta-D-glucopyranosyl- (1000->200)-beta-D-apiofuranosyldiphyllin	[31,32]
Schinus molle L.	Essential oils: hydrocarbon monoterpenes in the leaves and fruits (49.70% and 42.65%). The Essential Oil is characterized by the presence of γ-muurolene, γ-gurjunene, γ-cadinene, 10-epi-elemol, guaiol, and α-acorenol. Essential oil of leaves and fruits are β-pinene (10.36–5.44%), γ-terpinene (12.01–8.15%), limonene (22.94–18.49%), 10-epi-elemol (7.64–8.03%), γ-eudesmol (5.17–4.09%), and longifolene (7.67–8.48%).	[102]
	Essential oils: sesquiterpene and monoterpene hydrocarbons. The essential oil of leaves and fruits contains sabinene (48.63% and 51.74%) and limonene (10.20% and 16.98%), respectively, while bicyclogermacrene (18.12%) was leaves oil.	[103]
	Oil: α-phellandrene (35.86%), β-phellandrene (29.3%), β-pinene (15.68%), p-cymene (5.43%) and α-pinene (5.22%).	[33]
	Essential oil 98.55% (monoterpene hydrocarbons 79.69, monoterpenes oxygenated 11.75, sesquiterpenes hydrocarbons 3.69, sesquiterpenes oxygenated 1.83, Others 1.59)	[104]
	Sixteen components were identified (72.14% of the total components). The leaf extract contains Germacrene D (20.77%) and Beta-ceryophyllene (13.48%). Essential leaf oil contained 24 components: mainly delta-cadinene (11.28%) and alpha-cadinol (10.77%).	[105]
Matricaria chamomilla L.	120 chemical constituents, 28 terpenoids, 36 flavonoids. The coumarins are herniarin, umbelliferone, chlorogenic acid and caffeic acid (phenylpropanoids), apigenin, apigenin-7-O-glucoside, luteolin and luteolin-7-O-glucoside (flavones), quercetin and rutin (flavonols), naringenin (flavanone). α-bisabolol	[106,107]
	120 constituents. Essential oils composed of terpenoids, α-bisabolol and its oxides A and B, bisabolone oxide A, chamazulene, and β-farnesene. Phenolic compounds, including phenolic acids, flavonoids, and coumarins.	[43]

	The oil includes terpenoids, mainly sesquiterpenes, α-bisabolol, chamazulene, cis-β-farnesene. The major flavonoids are apigenin, theercetin, patuletin, and luteolin in concentrations of 16.8%, 9.9%, 6.5%, and 1.9%, respectively. Coumarins are herniarin and umbelliferone in 0.1%. Presence of 39% cinnamic acid derivatives such as ferulic acid and caffeic acid.	[108]
	A total of 22 phenolic compounds (7 hydroxycinnamic acids,13 flavonoids, and 2 phenolic acids). Nine main terpenoids, which is of the bisabolol chemotype.	[109]
	Sesquiterpenes, bisabolol oxide A, bisabolol oxide B, and bisabolone oxide. More than 200 compounds have been identified. Essential oil polyphenols, flavonoids like apigenin, luteolin, quercetin, patuletin, isorhamnetin and their derivatives, coumarins, peptides, and polyacetylene.	[110]
Valeriana sp.	No information about the species was found, but other species in the genera contain Valepotriates	[111,112]
Cucurbita pepo L.	Seeds contain five multiflorane-type triterpenoids: 3α--nitrobenzoylmultiflora-7:9(11)-diene-29-benzoate and 3α -acetoxymultiflora-7:9(11)-diene-29-benzoate.	[47]
	Phyto constituents belonging mainly to cucurbitosides, multiflorane-type triterpenoids, carotenoids, entkauranetype diterpene, and cucurbitaglycosides.	[113]
	Flavonoids, terpenoids, cardiac glycosides and cucurbitacins glycoside. Rich in nutritional components like carbohydrates, proteins, lipids and minerals.	[114]
	Seed and seed oil are a natural source of phytosterols, proteins, polyunsaturated fatty acids, antioxidant vitamins, carotenoids and tocopherols.	[115-117]
	p-aminobenzoic acid, γ-aminobutic acid, polysaccharides, peptides, proteins, carotenoids as lutein, lutein epoxide, 15-cis-lutein (central-cis)-lutein, 9(9')-cis-lutein, 13(13')-cislutein, α-carotene, β-carotene, violaxanthin, auroxanthin epimers, flavoxanthin, luteoxanthin, chrysanthemaxanthin, α-cryptoxanthin, βcryptoxanthin	[118]
	Saponins, cyanogenic glycosides and cardiac glycosides	[119]
Equisetum arvense L.	Flavonoids (kaempferol-3-O-sophoroside, kaempferol 3-O-(6”-O-malonyl-glucoside)-7-O-glucoside, kaempferol 3-O-sophoroside, quercetin 3-O-glucoside, apigenin, apigenin5-O-glucoside, luteolin, luteolin 5-O-glucoside, genkwanin 5-O-glucoside, isoquercitrin); phenolic acids (di-E-caffeoyl-meso-tartaric acid, mono-O- caffeoyl-meso-tartrate, methyl esthers of protocatechuic and caffeic acids. Barren sprouts: 5-caffeoylshikimic acid); phenolic petrosins (Onitin and oniti-9-O-glucoside); alkaloids (nicotine, palustrine, and palustrinine); triterpenoids (isobauerenol, taraxerol, germanicol, ursolic acid, oleanolic acid, betulinic acid); saponins (equisetonin), phytosterols (cholesterol,epicholestanol, 24-methylenecholesterol, isofucosterol, campesterol, and β-sitosterol).	[120]
	Phenolic acids, as di-E-caffeoyl-meso-tartaric acid, methyl esters of protocatechuic and caffeic acid, silicic acids, silicates, polyenoic acids, dicarboxylic acids and styryl-pyrone. Flavonoids: luteolin, quercetin, apigenin, isorhamnetin and kaempferol, quercetin and kaempferol (quercetin 3-glucoside and its malonyl esters). The steroid fraction is beta-sitosterol (60%), campesterol (32,9%), isofucosterol (5.9%), cholesterol (traces), Isoprenoid precursors, 3-oxo-alpha-ionol (spicy odor), (E,E)-pseudoionone (balsamic odor), benzenic derivatives, phenylethanal (hyacinth and lilac notes), 2-phenylethanol, traces of alkaloids (nicotine, palustrine and palustrinine).	[121]
	mono-O-caffeoyl-meso-tartrate, methyl esters of protocatechuic and caffeic acids. kaempferol. 3-O-sophoroside	[122]

	Phenolic petrosins: Onitin and oniti-9-O-glucoside kaempferol 3-O-(6″-O-malonylglucoside)-7-O-glucoside.	[55]
	Quercetin3-O-glucoside, apigenin, apigenin 5-O-glucoside	[123]
	Genkwanin 5-O-glucoside, isoquercitrin. Minerals: potassium (1.8%), calcium (1.3%), aluminium, sulphur, magnesium and manganese	[124]
Mimosa tenuiflora Poir.	Yuremamine was isolated from the stem bark	[58]
	Chalcones: kukulkan A (2′,4′-dihydroxy-3′,4-dimetoxychalcone); and kukulkan B (2′,4′,4- trihydroxy-3′-metoxychalcone)	[56]
	Thirteen compounds: eight type B proanthocyanidins, three alkaloids, a glycosylated flavonol, dihydrochalcone derivative.	[125]
	5,4′-dihydroxy-7-methoxyflavanone (sakuranetin), 5,4′-dihydroxy-7-methoxyflavone (genkwanin), 5,6,4′-trihydroxy-7-methoxyflavone (sorbifolin), 5,4′-di-hydroxy-7,8-dimethoxyflavone, and 5,7,4′-trihydroxy-3-methoxyflavone and the minor flavonols 5,7,4′-trihydroxy-6-methoxyflavonol, 5,6-dihydroxy-7,4′-dimethoxyflavonol and 5-hydroxy-7,8,4′-trimethoxyflavonol.	[126]
	Alkaloids, saponins, flavonoids, and terpenoids, mainly the indole alkaloid *N,N*-dimethyltryptamine	[127]
	Alkaloids, chalcones, flavonoids, indoles, terpenes, terpenoids, saponins, steroids, amino acids, glycosides, flavanols, phenols, lignoids, polysaccharides, lignins, salts and fatty esters	[128]
Salvia officinalis L.	α-thujone, 1,8-cineole, camphor, borneol and β-pinene	[129]
	Oils (1,8-cineole, camphor, α-thujone, β-thujone,borneol and viridiflorol).	[61]
	Monoterpenes (α-thujone, β-thujone,1,8-cineole, camphor); diterpenes (acid carnosic, carnosol, manool, rosmadial); triterpenes (acid oleanoic,ursolic acid); sesquiterpenos (α-humulene,viridiflorol) and phenolic acids (acid caffeic, acid vanillic, acid ferulic, rosmarinic acid) flavonoids (luteolin, apigenin, quercetin).	[60]
	The major constituents were 1,8-cineole (33.27%), β-thujone (18.40%), α-thujone (13.45%), borneol (7.39%) in oil	[33]
	Alkaloids, carbohydrate, fatty acids, glycosidic derivatives (cardiac glycosides, flavonoid glycosides, saponins), phenolic compounds (coumarins, flavonoids, tannins), poly acetylenes, steroids, terpenes/terpenoids, rosmarinic acid and luteolin-7-glucoside, phenolic acids such as caffeic acid and 3-Caffeoylquinic acid. Flavonoids like chlorogenic acid, ellagic acid, epicatecin, epigallocatechin gallate, quercetin, rosmarinic acid, rutin, and luteolin-7-glucoside, volatile components such as borneol, cineole, camphor, and thujone.	[130]
	120 components with a domination of borneol, caryophyllene, elemene, humulene, ledene, pinene, camphor, 1,8-cineole and thujone.	[131]
	Contains vital oils and other derivatives such as cinnamaldehyde, cinnamic acid and cinnamate. Cinnamon bark contains procyanidins (procyanidin A-type and B-type linkages) and catechins.	[63]
	The mayor compounds in cinnamon essential oil are cinnamaldehyde, camphor, cinnamyl-acetate, caryophyllene, trans α-bergamotene, caryophyllene oxide, linalool, geraniol, bornyl acetate, α-cubebene, γ-elemene, α-copaene, guaiol and eugenol.	[64]

Cinnamomum verum J. Presl.	Essential Oil from bark is cinnamaldehyde (56-78%), Essential Oil from leaves, eugenol (60-77%).	[132]
	Phenols (Eugenol, Pyrogallol); Acids (cinnamic acid, ferulic acid, caffeic acid, gallic acid, protocatechuic acid, oleic acid, p-hydroxybenzoic acid); monoterpenes(p-cymene,limonene,α-terpinene, α-pinene, camphene, camphor, (1,4)-cineole, β-pinene, β-phellandrene, α- phellandrene, 3-carene); diterpenes (cinnzeylanine, cinnezaylanol); sesquiterpenes (humulene, caryophyllene oxide, β-caryophyllene, α-muurolene, α-copaene,cedrene, α-tumerone, β-tumerone, α-cadinol, t-cadinol, calamenene,α-Ylangene); Flavonoids (A-type procyanidin dimer, b-type procyanidin dimer).	[133]
	Eugenol, cinnamaldehyde, cinnamyl acetate, copane and camphor.	[134]
Tilia americana var. *mexicana* (Schltdl.) Hardin	Several glycosides derived from quercetin and kaempferol, including tiliroside, isoquercetin, and quercetin-3-β-D-glucoside	[66]
	Hydrocarbons, esters, aliphatic acids, polyphenols and terpenoids, flavonoids (quercetin, kaempferol glycosides)	[71]
	Five flavonoids; tiliroside, quercitrin, rutin, quercetin glucoside, and kaempherol	[135]
	Polyphenolics of the phenylpropanoid pathway. The inflorescences contain high amounts of flavonoids (mostly in glycosidic form), catechins ((+)-catechin, (−)-epicatechin, procyanidin oligomers) and other phenylpropanoids (such as coumarins and cinnamic acid derivatives), polysaccharides and essential oil.	[136]
	From inflorescences and leaves flavonoid constituents: quercetin (20.09 ± 1.20 μg/mg and 3.39 ± 0.10 μg/mg), rutin (3.52 ± 0.21 μg/mg and 8.94 ± 0.45 μg/mg), and isoquercitrin (1.74 ± 0.01 μg/mg and 1.24 ± 0.13 μg/mg)	[69]
	Tiliroside (28.56), glucoside of quercetin (16.25), quercitrin (7.96), rutin (3.93), Kaempferol (2.83).	[137]
Aloysia citrodora Paláu	Four flavonoids, five caffeoyl derivatives, a phenylethanoid glycoside a hydroxycinnamic acid. Verbasoside, luteolin-7-O-diglucuronide, apigenin-7-O-diglucuronide, verbascoside, chrysoeriol-7-O- diglucuronide, isoverbascoside, forsythoside, eukovoside and martinoside, p-coumaric acid isorhamnetin-3-O-glucuronide.	[138]
	Two flavonoids vitexin and isovitexin. Essential oils included Limonene and 1,8-Cineole	[139,140]
	Essential oil: Neral, geranial, limonene, 1,8-cineole. Extract: Verbascoside and derivatives, flavonoids.	[73]
	The essential oil: oxygenated monoterpenes (49.7–53%), geranial and neral isomers.	[141]
	Oils: geranial (26.32–37.22%), neral (18.38–29.00%), and α-curcumene, spathulenol, caryophyllene oxide (8.66%).	[142]
Vitis vinifera L.	Constituents of the grape stem are two triterpenoid acids: oleanolic and betulinic acids; stilbenoid, daucosterol; E-resveratrol and its dimer E-ε-viniferin; gallic acid as a simple phenol; catechin and gallocatechin (flavanols); four 6′-O-acyldaucosterols; and five 1,2-di-O-acyl-3-O-β-D-galactopyranosyl glycerols.	[143]
	seeds contain gallic acid, p-coumaric, caffeic and ferulic acids.	[144]
	Polyphenols located in grapes, compounds in the seeds: (+)-catechins, and procyanidins dimers (B1-B5).	[74]

	Grape seeds: 40% fiber, 16% oil, 11% proteins, and 7% complex phenols such as tannins, gallic acid, p-coumaric, caffeic and ferulic acids. Roots: resveratrol, vitisins A & B and other stilbenoid compounds. Leaves: gallocatechin, catechins, procyanidins, epicatechins, hydroxybenzoic acid, syringic acid and coumarin. flavonoids and contain monomers, dimers, trimers, oligomers and polymers. The monomeric compounds include (+)- catechins, (-)-epicatechin, and epicatechin-3-O-gallate	[145]
Zingiber officinale Roscoe	6-gingerole, 6-shogaol, 6-paradol, zingerole and zerumbone	[146]
	Essential oils, phenolic compounds, flavonoids, carbohydrates, proteins, alkaloids, glycosides, saponins, steroids, terpenoids and tannin. Non-volatile pungent principles (gingerols, shogaols, paradols and zingerone). The volatile oil consists mainly of the mono- and sesquiterpenes; camphene, β-phellandrene, curcumene, cineole, geranyl acetate, terphineol, terpenes, borneol, geraniol, limonene, β-elemene, zingiberol, linalool, α-zingiberene, β-sesquiphellandrene, β-bisabolene, zingiberenol and α-farmesene. Zingiberol is component of ginger rhizome.	[147]
	6-Gingerol, 8-Gingerol, 10-Gingerol, 6-Shogaol, Caffeic Acid, Rosmarinic Acid, Syringic Acid, Amentoflavone, Ferulic Acid	[148]
	Phenolic compounds (gingerols, shogaols, and paradols), phenolic compounds (quercetin, zingerone, gingerenone-A, and 6-dehydrogingerdione); terpenes (β-bisabolene, α-curcumene, zingiberene, α-farnesene, and β-sesquiphellandrene), polysaccharides, lipids, organic acids, and raw fibers. Phenolic compounds in fresh ginger (6-gingerol, 8-gingerol, and 10-gingerol).	[149]
	A total of 43 compounds in essential oil, the most abundant components, respectively, were zingiberene (22.18%) and 1.8-cinéol (43.47%).	[150
	General compounds such as Fe, Mg, Ca, vitamin C, flavonoids, phenolic compounds (gingerdiol, gingerol, gingerdione and shogaols), sesquiterpenes, paradols. The pharmacological activities (6-gingerol, 6-shogaol, zingeron) besides other phenolics and flavonoids.	[151
	Volatile oil and gingerols are considered as the main components in rhizome	[152]
Larrea tridentata (DC.) Coville	Tannins, flavonoids, saponins, phytoestrogens and terpenes and bioactive molecules: ellagic acid, gallic acid, catechins, methyl gallate, cinnamic acid resorcinol, kaempferol, quercetin, nordihydroguaiaretic acid (NDGA), thymol and carvacrol.	[81]
	Phenolic, terpenoids, saponins, and odorous vinyl ketones. Abundant alkaloids and phenolic compounds such as chlorogenic acid. Nine compounds including Justicidin B and Beta peltain	[153]
	Phenolics, terpenoids, saponins and odorous vinyl ketones. 8–8′ linked lignan nordihydroguaiaretic acid.	[154]
	4,4′-dihydroxy-3-methoxy-6,7′-cyclolignan, 3,4-dihydroxy-3′,4′-dimethoxy-6,7′-cyclolignan. Lignans *meso*-dihydroguaiaretic acid, 3'-demethoxyisoguaiacin, 3'-demethoxy-6-*O*-demethylisoguaiacin, nordihydroguaiaretic acid, flavonoids 5,4′-dihydroxy-3,7,8-trimethoxyflavone; 5,8,4′-trihydroxy-3,7-dimethoxyflavone	[155]
	19 flavonoid aglycones, as well as several lignans, NDGA. Some glycosylated flavonoids, sapogenins, essential oils, halogenic alkaloids and waxes-contains about 0.1% of dry weight as volatile oils. volatile fraction 67 compounds in 90% of oils and 10% is a mixture of more than 300 constituents, mainly monoterpenoids and aromatic sesquiterpenoids.	[156]
